# Distribution of scoliosis in 2.22 million adolescents in mainland China: A population-wide analysis

**DOI:** 10.7189/jogh.14.04117

**Published:** 2024-07-19

**Authors:** Shuai Xu, Kexin Li, Linyu Jin, Yanhui Dong, Yan Liang, Chenjun Liu, Peihan Wang, Zhuran Zhao, Yixuan Wang, Chen Guo, Zhenbo Wang, Haiying Liu

**Affiliations:** 1 Department of Spinal Surgery, Peking University People’s Hospital, Peking University, Beijing, China; 2 Institute of Geographical Sciences and Natural Resources Research, Chinese Academy of Sciences, Beijing, China; 3 Institute of Child and Adolescent Health, School of Public Health, Peking University, Beijing, China; 4 Department of Paediatrics, Peking University People’s Hospital, Peking University, Beijing, China; 5 Department of Pain Medicine Centre, Peking University Third Hospital, Peking University, Beijing, China

## Abstract

**Background:**

The characteristics of scoliosis afflicting school children and adolescents in mainland China are still unclear. Therefore, we conducted a systematic review to estimate scoliosis’s prevalence and characterise its distribution in China.

**Methods:**

We screened PubMed, Scopus, WanFang, China National Knowledge Infrastructure, National Science and Technology Library, and WeiPu databases for mainland China articles published between 1 January 1980 and 31 October 2022. Among them, we identified population-wide scoliosis studies in school children and adolescents. The main outcomes were the positive rate of primary screening and the prevalence of final screening. Primary screening mainly included general examination with/without the forward bending test in school. The final screening entailed clinical diagnosis by Röntgen radiation in a hospital (based on primary screening). A meta-analysis of scoliosis distribution by gender was performed to calculate the odds ratios (ORs) and 95% confidence intervals (CIs). Further, we analysed the distributions of scoliosis by age, region, aetiological type, and severity of curvature, in addition to the correlation between its prevalence and altitude or latitude.

**Results:**

77 studies with 2 224 320 participants were included. The positive rate through primary screening was 3.97%, whereas the prevalence of scoliosis at final screening was 1.20%. Analysing the data revealed a higher prevalence of scoliosis in girls (OR = 1.57; 95% CI = 1.38–1.81). The age-wise peak rate of scoliosis was 15–16 years (1.07%) in boys and 13–14 years (1.42%) in girls. The mean prevalence of scoliosis was 1.07% in the western region, 1.54% in the central, and 1.35% in the eastern. Scoliosis prevalence was not correlated with either altitude or latitude. The prevalence of idiopathic and congenital scoliosis was 1.18 and 0.03%. Among all subjects with scoliosis, 79.10 and 16.80% had mild and medium disease severity.

**Conclusions:**

According to this comprehensive study using data sets of scoliosis in adolescents across mainland China, the mean prevalence of scoliosis is 1.20%, yet 1.57 times higher in girls than boys, and is most prevalent in the middle region. Overall, scoliosis in adolescents could pose a burden to public health in mainland China.

**Registration:**

PROSPERO CRD42021231987.

Scoliosis is a complex three-dimensional torsional deformity in the spine and torso, with an established diagnostic criterion of a Cobb angle >10 degrees measured by Röntgen radiation (x-ray) [1]. Yet scoliosis in school children and adolescents is sometimes overlooked without periodic screening. Thus, treatment is usually recommended for them in the progressive period of the disease not only to improve their deformed appearance but also to mitigate cardiopulmonary dysfunction or address psychosocial disorders [2]. Together, this can increase the financial burden of caregivers by 7–27% [3]. In recent decades, screening students in schools for scoliosis has been widely carried out for timely monitoring and controlling of scoliosis [4–6].

As officially reported, the prevalence of scoliosis in primary and secondary schools in mainland China ranges from 0.11–2.64%, while a review done in 2014 determined a general prevalence of 1.02% for mainland China [7]. However, the characteristics of scoliosis remain uncertain [8,9]. Hence, because positive cases are overlooked in previous reviews, the positive cases identified by primary screening in school should also be considered, aside from those based on an x-ray diagnosis [6].

Given mainland China’s vast territory, it is meaningful, though challenging, for the government to implement the necessary screening and protective measures to address scoliosis as a potential public health concern [1,10,11]. The idea is to determine the nationwide prevalence and distribution of scoliosis. In recent years, a series of scoliosis screening programmes have been carried out in various regions, and though these harbour much interregional heterogeneity, these studies nonetheless offer the possibility of integrating their data. Accordingly, we conducted a systematic review to estimate the prevalence of scoliosis and characterise its distribution in mainland China.

## METHODS

### Search strategy

The study’s protocol was approved by the ethics committee of our institution and is available in PROSPERO (CRD42021231987). We collected published studies from mainland China from PubMed, Scopus, WanFang, China National Knowledge Infrastructure, China National Science and Technology Digital Library, and WeiPu databases. The search period was from inception until 31 October 2022. According to our research strategy, we used keywords (namely scoliosis, school, epidemiological survey, screening, prevalence, incidence, and mainland China) to screen for potentially relevant publications (Table S1 in the **Online Supplementary Document**). Two reviewers independently screened all the studies for their eligibility. Any discrepancies were resolved through a third reviewer to reach a consensus. If more than one study contained the same population with similar outcomes, only that study reporting newer and more specific information was selected.

### Inclusion and exclusion criteria

We applied the following inclusion criteria: 1) the original population-wide research was conducted within mainland China, 2) the diagnosis of scoliosis was based on a general examination using the forward bending test (FBT), angle of trunk rotation (ATR), or radiography, 3) there was a reported positive rate by primary or secondary screening, or evidence of definitive scoliosis by a Cobb angle >10 degrees confirmed in a third evaluation (radiographic), and 4) if more than one article reported the same cohort with complementary data, all the articles were included. Notably, the primary screening methods mainly included general examination by inspection and palpation, the FBT, or the detection of ATR using a scoliometer tool conducted in school to screen positive subjects. The final screening (or third screening) refers to the procedure for obtaining a clinical diagnosis of scoliosis by x-ray in the hospital, applied to positive subjects identified by primary screening. The exclusion criteria were: 1) duplicated publications, 2) case studies, reviews, comments, or letters, and 3) studies with insufficient or nonspecific data.

### Data extraction and quality assessment

Two reviewers independently extracted the pertinent information from eligible studies, with any discrepancies resolved by discussing with a third reviewer to reach a consensus. The following data were extracted from each study: 1) author’s name, publication year, and province or city, 2) the screening sample of participants and their age, 3) methods used and positive rate outcome of primary and secondary screening, 4) prevalence of scoliosis, and 5) types and severity of scoliosis.

Two investigators independently graded each eligible article by applying the modified quality methods of population-wide studies in accordance with the Strengthening the Reporting of Observational Studies in Epidemiology (STROBE) Statement [12]. STROBE statement refers to the list of items of observational studies that should be reported in their cross-sectional designs. This amounts to 22 items covering six normative aspects – title and abstract, introduction (background/principles, purpose), methods (research design, research settings, participants, variables, data sources/measurements, bias, sample size, quantitative variables, statistical methods), results (participants, descriptive data, outcome data, main results, other analyses), discussion (key results, limitations, explanations, generalisability), and other information (funding sources) (Table S2 in the **Online Supplementary Document**).

### Data analysis

We calculated the distribution of scoliosis prevalence by gender through a meta-analysis, for which prevalence was either extracted as reported from the studies or calculated from their original data. Scoliosis or not were considered dichotomous variables and reported as odds ratios (ORs) with their 95% confidence intervals (95% CI) obtained by the Mantel-Haenszel method. To estimate the heterogeneity of studies, *I^2^* was used, where an *I^2^*<50% indicated low heterogeneity, with results then pooled using a fixed-effects model. Conversely, an *I^2^*>50% indicated high heterogeneity, with results pooled using a random-effects model [12]. The entire meta-analysis contained both the positive rate from primary screening and the prevalence from third screening. Furthermore, a subgroup meta-analysis was conducted by stratification according to mainland China’s three geographic regions (eastern, central, and western) to assess the spatially distributed prevalence of scoliosis.

Because idiopathic scoliosis is the most common type of scoliosis, it is thus clinically significant, so we included the description of idiopathic scoliosis in our study. In addition, the age distribution of subjects with scoliosis (7–18 years old) was examined for both their primary screening and final diagnosis, and this further distinguished them among eastern, central, and western regions. Lastly, we also characterised the distribution of scoliosis by its aetiological types and severity. The prevalence of scoliosis was also tested to determine whether it was correlated with altitude or latitude.

All meta-analyses were conducted using Review Manager, version 5.3 (Cochrane Collaboration, Oxford, UK). The results were considered statistically significant if their two-sided *P*-values <0.05. Each meta-analysis followed the Preferred Reporting Items for Systematic Reviews and Meta-Analyses statement guidelines (Table S3 in the **Online Supplementary Document**).

## RESULTS

### Search results

**Figure 1:**
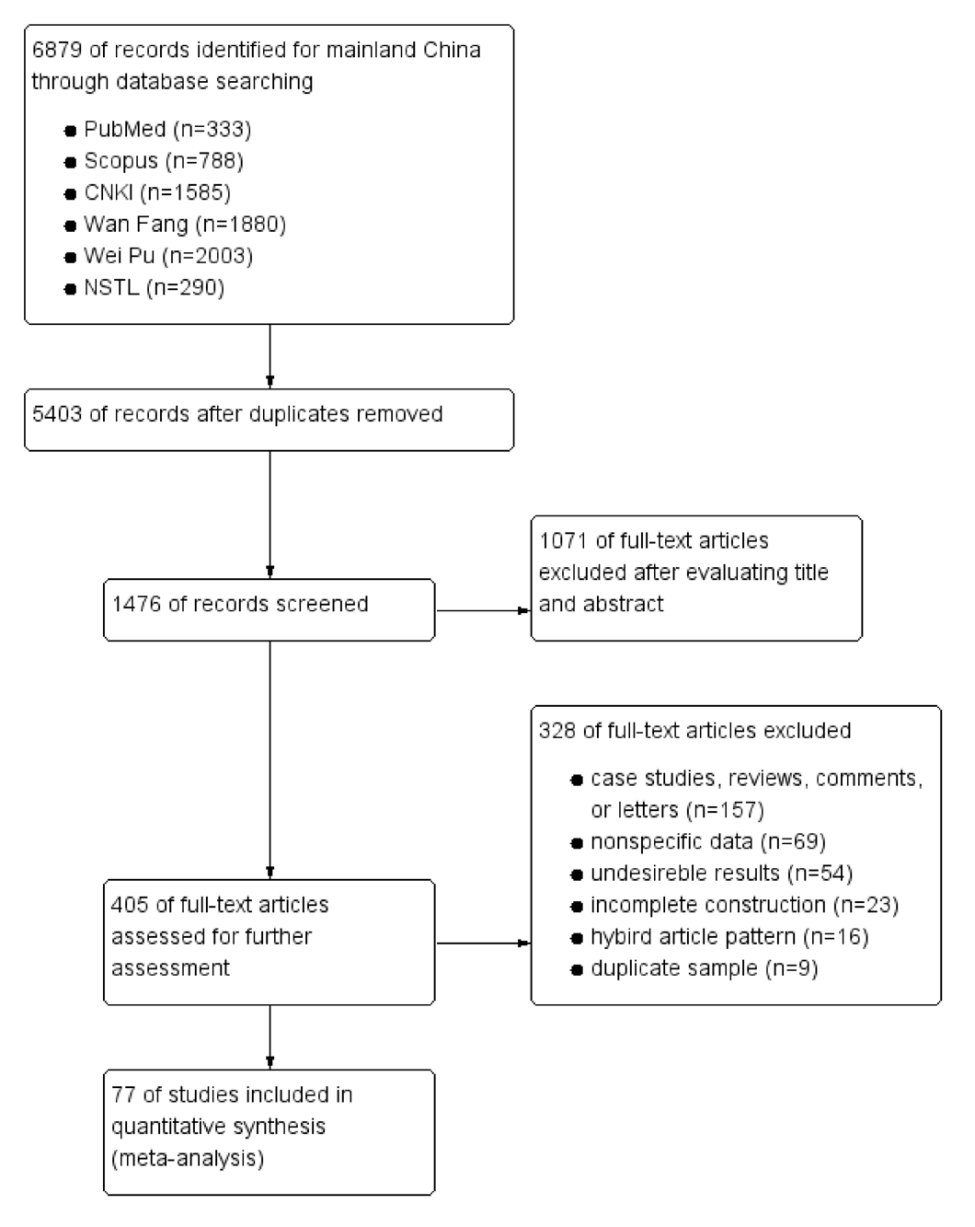
Flow diagram presenting screening of eligible studies.

Initially, 6879 studies were identified for mainland China, of which 77 studies with 2 224 320 participants were deemed eligible for the meta-analysis [6,13–88] (**Figure 1**, **Table 1**). The search periods spanned 1983–2022, while the participants ranged from five to 20 years old. There were five studies for Guangzhou and four for Beijing, Shanghai, and Shenzhen, respectively. Guangdong has the most cities (11 cities) that implemented primary and third screening. Eight studies only included primary screening, while seven included both primary and secondary screening. The most frequent primary screening methods were physical examination (93.50% of studies) and ATR measurement with the FTB (29.90%). However, radiographic assessment was directly applied in Lanzhou, and Lhasa was free of primary screening. The most common secondary screening method was the Moiré pattern, and 80.50% of the studies mentioned a third screening (Table S4 in the **Online Supplementary Document**). The quality assessments of the included articles are according to the STROBE statement (Table S5 in the **Online Supplementary Document**).

**Table 1:** Details of the included studies

Studies	Province	City	Participants (n)			Age in years	First screening (n)*	Second screening (n)*	Third screening (n)*			Idiopathic scoliosis (n)
			**Total**	**Boys**	**Girls**				**Total**	**Boys**	**Girls**	
Qiu 2022 [13]	Jiangsu	Wuxi	18 562	9135	9427	7–18	578	303	234	58	149	207
Chen et al. 2021 [16]	Hebei	Shijiazhuang	1426	737	689	5–8	23 804					
Sun et al. 2021 [17]	Guangdong	Guangzhou	2121	1100	1021	NA		256	33	11	22	32
Li et al. 2021 [15]	Sichuan	Leshan	1465	100	1365	5–16	3426		534	246	288	480
Wen et al. 2021 [14]	Sichuan	Mianyang	8176	4185	3991	7–18	672	233	81	40	41	
Cai et al. 2021 [18]	Guangdong	Chaozhou	5497	3018	2479	6–12		265				
Ding et al. 2020 [19]	Henan	Zhengzhou	6594	9317	9201	6–18			126	55	71	126
Yang et al. 2020 [20]	Guangdong	Shenzhen	595 057	324 932	270 125	6–19	1543	263	136			136
Xia et al. 2019 [21]	Shanghai	Shanghai	3913	2077	1836	7–15	2105		1238	649	769	649
Zeng 2019 [22]	Guangdong	Huizhou	104 088	58 542	45 546	7–17	156	141	123	54	69	107
Wang et al. 2018 [23]	Beijing	Beijing	25 097	12 932	12 165	13–18	50		23			23
Wei et al. 2018 [24]	Hubei	Yichang	3483	1797	1686	9–15	428	228				213
Li et al. 2018 [25]	Guangdong	Shenzhen	15 247	7446	7801	11–16	520		102			102
Du et al. 2018 [26]	Guangdong	Shantou	12 881	6626	6255	11–17	2202		200	71	129	195
Huang et al. 2018 [27]	Guangdong	Zhongshan	41 258	21 432	19 916	12–18			59			59
Deng et al. 2018 [28]	Sichuan	Ganzi	5126	2745	2381	12–17	236		29	8	21	
Wang et al. 2018 [29]	Yunnan	Kunming	784	315	469	9–16	101					
He et al. 2018 [30]	Qinghai	Xining	13 121	6553	6568	12–16	561	268	151			142
Tang et al. 2017 [31]	Shanghai	Shanghai	5327	2748	2579	11–13			52	14	38	52
Miao et al. 2017 [32]	Jiangsu	Wuxi	67 322	36 888	30 434	10–17	442		172	68	104	
Nie et al. 2017 [33]	Zhejiang	Lishui	3100			8–15	282					11
Li et al. 2017 [34]	Henan	Luohe	14 326	7231	7095	5–10			154	69	85	154
Deng et al. 2017 [35]	Hubei	Xiangyang	2504	1111	943	7–17	7912		5140	1255	3870	5125
Hu et al. 2017 [36]	Guangdong	Shenzhen	19 870	11 860	8010	12–18		218				
Han et al. 2017 [37]	Gansu	Lanzhou	4993	2395	2985	15–20	1012	708	360	146	214	351
Du et al. 2016 [38]	Shanghai	Shanghai	6824	3477	3347	6–17			166	41	120	161
Zheng et al. 2016 [39]	Jiangsu	Wuxi	11 024	5908	5116	6–13	375		88	42	46	42
He et al. 2016 [40]	Fujian	Quanzhou	21 415	11 491	9924	10–18	1121	789	393	160	233	
Fan et al. 2016 [6]	Guangdong		99 695	50 584	49 111	10–19			10 831			10 831
Huang et al. 2016 [41]	Yunnan	Kunming	13 802	6622	7180	6–19	420		250	109	141	240
Chen et al. 2016 [42]	Shaanxi	Xi'an	27 890	14 809	13 081	7–18	175		85	31	54	81
Ke et al. 2015 [43]	Jiangsu	Zhenjiang	15 667	7944	7723	12–18	571		89	41	48	89
Ma et al. 2015 [44]	Hainan	Sanya	6952	3750	3202	10–16	418	191	112	49	63	
Chen et al. 2015 [45]	Shaanxi	Xi'an	30 742	15 898	14 844	7–18	408	213	156	67	89	150
Yu et al. 2014 [46]	Guangdong	Guangzhou	29 532	12 337	17 195	7–18	399	175	122	66	70	
Zhao et al. 2014 [47]	Guangdong	Guangzhou	8351	4211	4140	7–15	55	15	10	3	7	
Ren et al. 2014 [48]	Sichuan	Zigong	17 348	9757	7591	7–17	1240	518	423	196	227	368
Wang et al. 2013 [49]	Zhejiang	Wenzhou	18 154	9319	8835	7–18			211	31	180	192
Ke et al. 2012 [50]	Guangdong	Foshan	18 798	9644	9154	7–15	851		49			
Chen et al. 2012 [51]	Guangdong	Yangjiang	19 646	10 661	8985	7–16			134			129
Zhang et al. 2011 [52]	Inner Mongolia	Huhhot	1260	630	630	7–13			41	7	34	38
Liu et al. 2011 [53]	Heilongjiang	Harbin	24 362	12 222	12 140	6–16	911	413	335	147	188	311
Huang et al. 2011 [54]	Guangdong	Guangzhou	30 124	15 384	14 758	7–20		5299				
Tang et al. 2011 [55]	Guangdong	Shenzhen	40 579			6–15	181	181	98			94
Li et al. 2011 [56]	Guangdong	Zhongshan	44 058			7–19	197		64	23	41	62
Chen et al. 2010 [57]	Liaoning	Jinzhou	12 257	6324	5933	7–16	877	423	234	93	141	229
Lu et al. 2010 [58]	Heilongjiang		17 525	9017	8508	7–15	476	305	158	78	80	153
Yu et al. 2010 [59]	Fujian	Xiamen	116 907	63 544	53 363	6–20	1894		184	75	109	175
Du et al. 2010 [60]	Guangdong	Foshan	13 247	7215	6032	>10	70					
Dong et al. 2009 [61]	Jiangxi	Nanchang	10 119	5444	4675	9–15			64			58
Zhou et al. 2008 [62]	Fujian	Quanzhou	32 280	17 212	15 068	7–20	331					
Zhang et al. 2008 [63]	Fujian	Quanzhou	21 112	11 336	9776	7–18	1389	607	343	164	179	321
Sun et al. 2008 [64]	Guizhou	Liupanshui	17 555	9465	8090	9–16	321	116	72	39	33	67
Wang et al. 2007 [65]	Beijing	Beijing	57 581			5–20	274	93	65	34	31	63
Yu et al. 2006 [66]	Zhejiang	Hangzhou	7138	3671	3467	10–14	827	315	251	110	141	225
Cheng et al. 2006 [67]	Shaanxi	Xi'an	25 725	13 875	118 50	7–15	242		17	9	8	9
Liang et al. 2005 [68]	Guangdong	Zhaoqing	8210	4159	4051	4–7	1765	857	653	287	366	633
Gao et al. 2004 [69]	Jiangsu	Nantong	8652	4395	4257	7–15			19	9	10	
Meng et al. 2003 [70]	Hebei	Langfang	16 658	8501	8157	7–17	886	453	361	158	203	350
Zhang et al. 2003 [71]	Hainan	Haikou	8198	4423	3775	7–16	75		13	3	10	
Liu et al. 2002 [72]	Guangdong		87 546	46 952	40 594	7–18	6					
Liang et al. 2002 [73]	Tibet	Lhasa	5737	2747	2990	15 · 6	107	90	74	29	45	72
Li et al. 2001 [74]	Guangdong	Guangzhou	33 798	17 644	16 154	7–15	613	187	112	59	53	107
Li et al. 1999 [75]	Guangdong	Shaoguan	21 859	11 397	10 462	7–15	100					
Li et al. 1999 [76]	Guangdong	Zhongshan	18 329	10 116	8213	7–15	327	213	104	47	57	101
Wang et al. 1998 [77]	Guangdong	Zhuhai	13 560	7069	6491	10–19	188					
Wang et al. 1996 [78]	Beijing	Beijing	21 759			8–14	902		231			202
Zhao et al. 1996 [79]	Shanghai	Shanghai	10 073	5230	4843	6–15	563	487	487	207	280	
Yu et al. 1995 [80]	Tianjin	Tianjin	8263	4399	3864	6–16	285		158			153
Ma et al. 1995 [81]	Shanxi	Changzhi	24 130	12 547	11 583	7–18	1794		347	160	187	313
Jiang et al. 1994 [82]	Tianjin	Tianjin	37 003	18 849	18 154	6–12	668		422	180	242	
Chen et al. 1990[83]	Shandong	Qingdao	26 980	11 187	10 246	11–19	1544	130	41			
Cao et al. 1989 [84]	Jilin	Yanbian	10 329			7–18	826					
Zhang et al. 1988 [85]	Beijing	Beijing	20 418	10 283	10 135	7–15	1222	612	213	86	127	
Tan et al. 1987 [86]	Guangxi	Nanning	33 079	18 198	14 881	7–12	276					
Wang et al. 1985 [87]	Jiangsu	Nanjing	2567	1336	1231	NA	344					
Pin et al. 1985 [88]	Hunan	Changsha	8165	4202	3963	6–15	790		171	74	97	167

NA – not available

*First screening indicates the positive number by the primary screening, and so on for the second and third screening. The primary screening was recorded when it was mentioned in the original study. Although it might be the first/sary screening method in the original article, the radiographic examination was uniformly seen as the third screening method in this study; hence, the first/sary screening data may be blank.

Sixty-two studies (24 provinces and 42 cities) provided data from primary screening. The province and city with the highest positive rate were Guizhou (10.80%) and Jiangsu-Nanjing (13.40%), while Guangxi and Guangdong-Shaoguan had the lowest prevalence (0.84 and 0.49%). A total of 62 studies (23 provinces and 39 cities) reported the determined prevalence of scoliosis, the highest being Shanghai (3.04%), while Tibet and Shandong-Qingdao had the lowest prevalence (0.33 and 0.15%).

The positive rate from primary screening was 3.97% (n = 74 488/1 874 610) and 2.12% (n = 16 324/771 268) from secondary screening, while the prevalence from the third screening was 1.20% (n = 16 727/1 402 121), with a prevalence of 1.80% in girls and 0.95% in boys. In addition, there was a linear relationship between the scoliosis prevalence and the positive rate from primary screening (*P* < 0.001), whose regression equation was:

positive rate of first screening = 1.16 × prevalence +2.80 (coefficient of determination (*R^2^*) = 0.231).

### Meta-analysis for the distribution of scoliosis by gender

**Figure 2:**
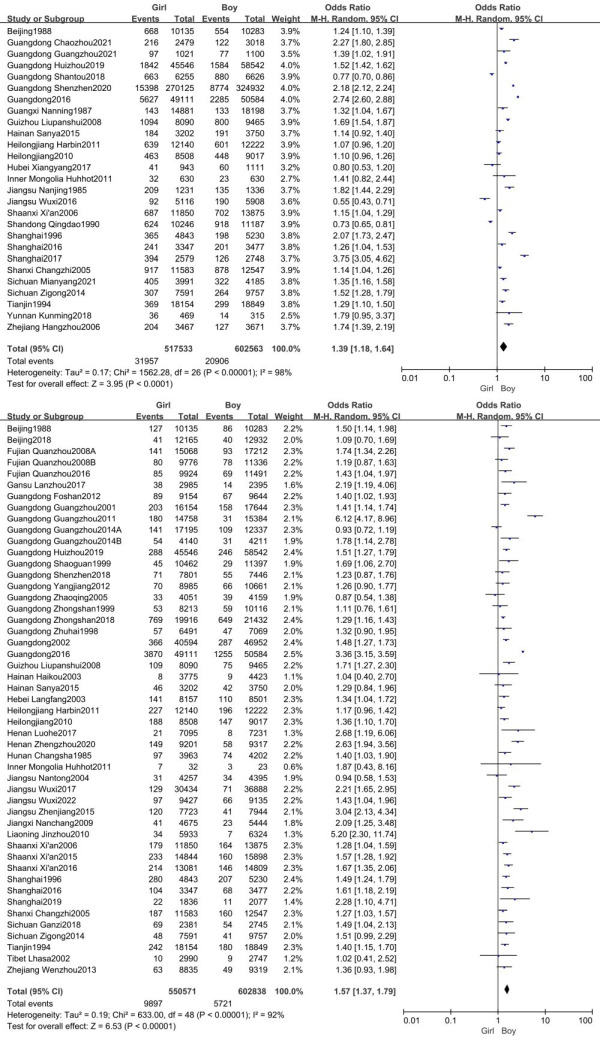
Forest plot for the total meta-analysis of scoliosis prevalence by gender. **Panel A.** Forest plot of the positive rate from primary screening. **Panel B.** Forest plot of the prevalence of scoliosis from final screening.

For the outcome of primary screening, 27 studies (n = 1 120 096 participants) were included in this meta-analysis. The positive rate of girls exceeded that of boys based on the random-effects model (OR = 1.39; 95% CI = 1.18–1.64, *P* < 0.001, *I^2^* = 98). A total of 49 studies (n = 1 153 394 participants) reported the prevalence of scoliosis, which was higher in girls than boys (OR = 1.57; 95% CI = 1.37–1.79, *P* < 0.001, *I^2^* = 92) (**Figure 2**).

For the subgroup geographical analysis of primary screening data, five studies corresponded to mainland China’s western region by gender, five to central, and 17 to eastern. All subgroups showed significant differences by gender (OR = 1.44, 95% CI = 1.20–1.72; OR = 1.11, 95% CI = 1.04–1.18; and OR = 1.47, 95% CI = 1.19–1.81). For the final screening, there were eight studies in the western, eight in the central, and 33 in the eastern regions. All showed statistical differences by gender (OR = 1.53, 95% CI = 1.38–1.70; OR = 1.58, 95% CI = 1.27–1.97; and OR = 1.58, 95% CI = 1.30–1.86) (Figure S1 in the **Online Supplementary Document**).

### Scoliosis distribution by age

A total of 34 studies reported an age-group distribution for scoliosis. Seven studies mentioned primary screening information, three presented second screening, and 27 were third screening. 12 studies found a passing description instead of specific data. Overall, five and 18 studies provided specific age-group data by gender.

**Figure 3:**
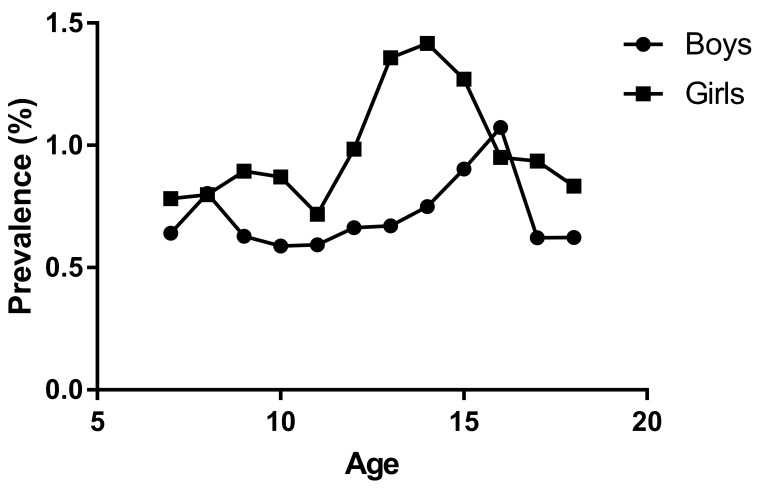
Distribution of the prevalence of scoliosis in various age groups by gender.

For the primary screening outcome, the positive rate of scoliosis tended to increase with age for either gender. It peaked among boys in the age group of 17 years old (mean (x̄) = 11.90%), while among girls, the apex was reached in the group of 16 years old (x̄ = 9.60%). For the outcome of the third screening by x-ray, the rate of scoliosis showed two peaks in growth in the group from seven to 10 years old and from 12–16 years old. The apex ended in the group of 14 years. Specifically, the peak was in the 15–16 age group (1.07%) among boys while in the 13–14 age group (1.42%) among girls (**Figure 3**, Table S6 in the **Online Supplementary Document**).

### Scoliosis distribution by region

**Figure 4:**
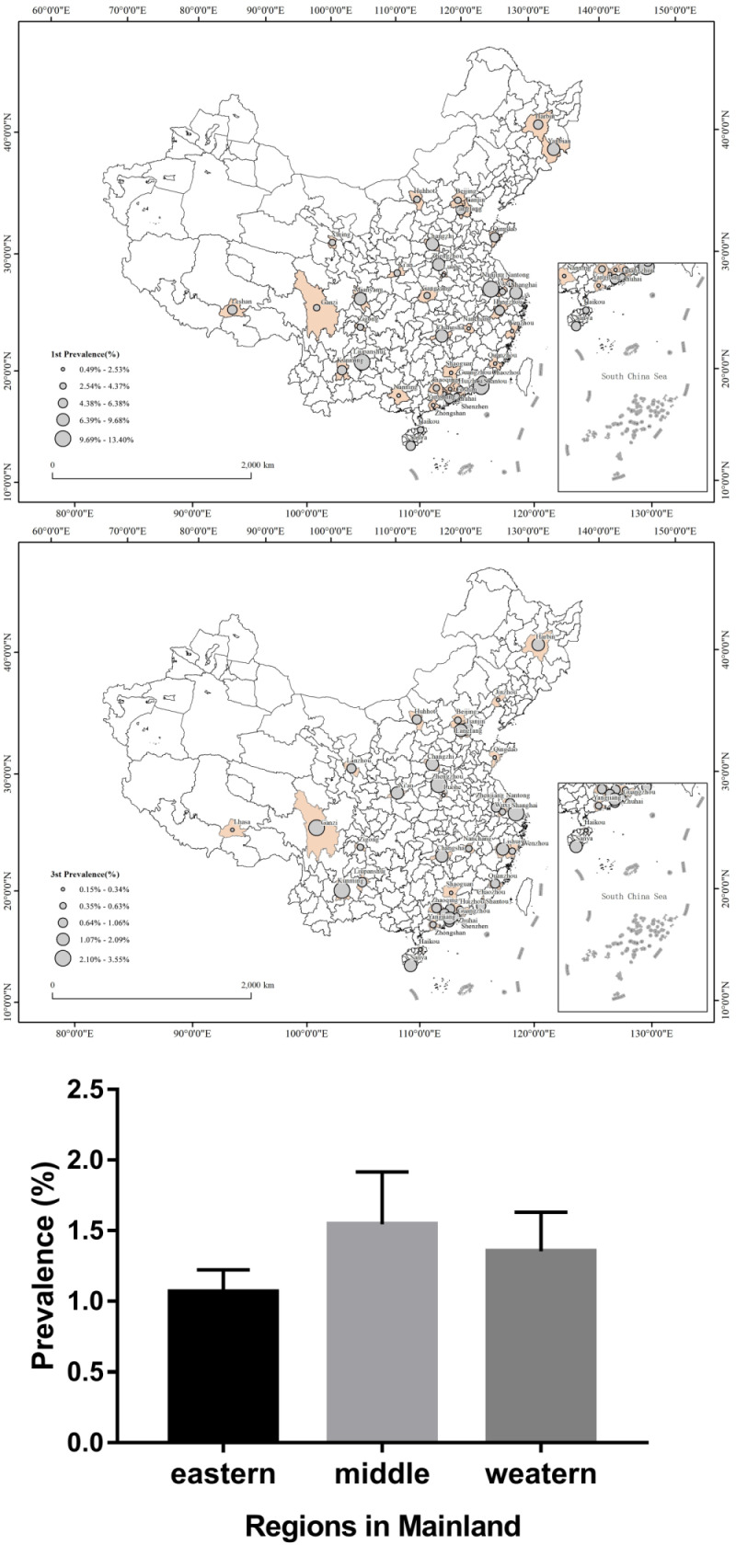
Geographical distribution of the prevalence of scoliosis. **Panel A.** Map showing the spatial distribution of the positive rate from primary screening. **Panel B.** Map showing the spatial distribution of prevalence of scoliosis from final screening. **Panel C.** Prevalence of scoliosis in mainland China’s eastern, central, and western regions.

Concerning primary screening, 10 studies were done in the western region, 10 in the central region, and 42 in the eastern region. The x̄ positive rate were 5.02% (n = 7429/147 932), 5.64% (n = 6728/119 314), and 3.75% (n = 60 331/1 607 364). For the third screening, there were eight studies in each western and central region and 46 in the eastern region. The x̄ prevalence was 1.07% (standard deviation (SD) = 1.05, n = 13 617/1 177 088); 1.54% (SD = 1.05, n = 1613/106 481), and 1.35% (SD = 0.83, n = 1586/135 900) (**Figure 4**, panels A–C). Hence, according to the primary and third screening, scoliosis was most pronounced in the central region. However, there was no relationship between scoliosis prevalence and either latitude (*P* = 0.456) or altitude (*P* = 0.733), and likewise for the positive rate from primary screening.

### Scoliosis distribution by aetiological types and disease severity

A total of 50 studies (21 provinces) included the outcomes of idiopathic scoliosis, which had a prevalence of 1.18% (n = 13 529/1 150 865). Furthermore, 34 studies referred to other types of scoliosis, namely congenital (0.037%) or neuromuscular scoliosis (0.0090%), as well as others (0.0067%) (Figure S2, Table S7 in the **Online Supplementary Document**).

**Figure 5:**
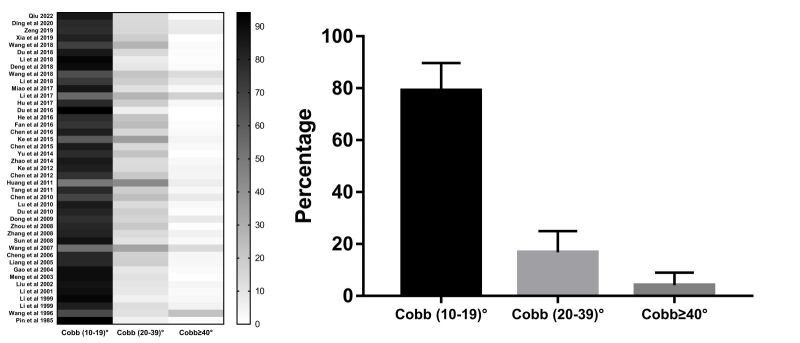
Distribution of the severity of scoliosis in terms of mild, medium, and severe cases. **Panel A.** The distribution of all three severity levels in the included studies. **Panel B.** The pooled distribution of scoliosis severity.

The severity of scoliosis was classified in 43 studies, where, among all patients, mild scoliosis (Cobb angle = 10–19) was most common, at 79.10%, followed by medium scoliosis at 16.80% (Cobb angle = 20–39) and severe scoliosis at 4.13% (Cobb angle >40) (**Figure 5**, panels A–B, Table S7 in the **Online Supplementary Document**). Three levels of severity were similar in proportion among regions (western vs. central region *P* = 0.842, western vs. eastern region *P* = 0.684, and central vs. eastern region *P* = 0.332). Their relationship to altitude was also not significant (western vs. central region *P* = 0.839, western vs. eastern region *P* = 0.608, and central vs. eastern region *P* = 0.672).

## DISCUSSION

Routine scoliosis screening in school is being widely carried out, yet some countries still discourage scoliosis screening because of its poor cost-effectiveness [11,89]. Research on scoliosis screening in mainland China seems inadequate in this public health context, with scattered data from various regions, inconsistent planning, and limited data synthesis [6]. Given mainland China’s huge landmass and population, there is likely a large base of school children and adolescents with poor posture or scoliosis. Hence, it is significant to clarify the spatiotemporal distribution of scoliosis.

A systematic review performed in 2014 concluded that the prevalence of scoliosis across mainland China was 1.02% among primary and secondary school students [7]. Our study updated the data for mainland China from 1983 to 2022 and separately identified the positive rate of scoliosis from primary screening and its prevalence by x-ray, which enabled us to provide a theoretical basis for on-site implementation. The difference in estimated prevalence between the 2014 review and our study is likely due to the former’s hybrid outcome from primary and final screening with enlarged reporting bias, while a stratification by screening stage was emphasised here. The outcomes could be adjusted by including a close twice sample and studies of the previous study [7], which could be more valuable and representative of homogeneous studies. Further, unlike similar prior studies, we investigated the multi-dimensional distribution of scoliosis by gender, age, region, and aetiological type for the first time.

Although the positive rate obtained via preliminary screening is not the actual prevalence of scoliosis, it was considerable [9,90,91]. First, the population we examined contained students with paraspinal muscle unevenness, imbalanced posture, and non-structural or potential structural scoliosis, all of whom might benefit from professional advice and regular follow-ups, the key goal of performing a nationwide investigation. In addition, the high prevalence of 3.97% from preliminary screening could provide the data evidence for use in sample-size calculations for further screening and policy-making concerning scoliosis [7,9]. Its definitive prevalence in children and adolescents was 1.20%, so we estimate that it presently affects three million children and adolescents in mainland China, which probably leads to considerable government health expenditures and burdens on their families [3,92].

In general, compared with boys, a greater proportion of girls had severe scoliosis and earlier peak age of scoliosis, especially for idiopathic types. Yet the cause of Adolescent idiopathic scoliosis (AIS) is multifactorial, and much research on the aetiology has focused on connective tissue abnormalities, nutritional deficiency, and genetic factors [3,90,93]. It is known, however, that both AIS and central precocious puberty are more common in girls than in boys and that scoliosis progression is linked to their growth spurt. A recent study reported a higher AIS prevalence for girls with central precocious puberty than another group and suggested that gonadotropin-releasing hormone treatment for central precocious puberty may have a suppressive effect on the progression of AIS [94]. In another study, Wise et al. [95] emphasised that AIS is remarkable in its sexual dimorphism, finding that girls face a 5-fold greater risk of progressive deformity than boys. Using pathway-level analyses of genetic data sets, the authors also highlighted the role of cartilage biogenesis and intervertebral disc development in AIS susceptibility. In addition, the large heterogeneity we uncovered in our scoliosis meta-analysis could arise from the significant prevalence estimates reported by various studies, the huge sample of cross-sectional studies with accurate estimation, and the single arm meta-analysis performed in this study, all of which also implicates the value of pursuing uniformed nation-wide screening.

Zhang and Grivas et al. [7,96] reported that the prevalence of scoliosis increased with higher latitudes, probably because of the varying lifestyles at differing geographical latitudes. However, we did not find evidence that obvious features of scoliosis vary across latitude and altitude, but this should be further explored by rigorous epidemiological surveys. The disparity among included studies likely arose from their various classifications, methods of sample selection, precision of radiological devices, specialisation of the investigator, and local policies for students’ health.

The prevalence of scoliosis was greater in the central region of mainland China than in its other two regions. Still, the reason for that requires further investigation and verification. Apart from genetic susceptibility, it is presumed that the aggravation of spinal deformity is caused by the imbalanced tension of paraspinal muscle, asymmetrical biomechanics of the intervertebral disc and facet, inadequate exercise, and poor nutrition. In contrast to central and western regions, the students in the eastern region might have access to better health care, given the higher socio-demographic index. Moreover, in the western region, there is a higher school dropout rate, possibly leading to scoliosis deformity being underestimated in its smaller sample of school children and adolescents.

There was an obvious relationship between the primary screening’s positive rate and the prevalence of scoliosis (correlation coefficient (r) = 0.481), which suggests that the primary screening could be used to roughly predict the diagnosis rate with certain reliability [97,98]. More specifically, almost all cases of scoliosis diagnosed by x-ray were of structural deformity. It is usually dominated by a complex genetic susceptibility, and the prevalence of this clinical spectrum should remain stable in the long term. Hence, although studies were limited to 1980–2020, the spatiotemporal distribution model could still be generalised as a simple cross-sectional spatial distribution model. In addition, the map for the primary screening’s positive rate was similar to that obtained for the third screening’s prevalence, bolstering the dependability and repeatability of using primary screening in school.

This study summarised almost all available research on the scoliosis screening programme for school children and adolescents in mainland China. Despite interregional inconsistency in many aspects, there is no doubt that this work fills a major knowledge gap in the present scoliosis situation in mainland China. Using the largest sample of subjects from mainland China, this study further identified the prevalence of scoliosis, its huge patient base, and the characteristics of its regional distribution. This work supplements the evidence for drawing official attention to children’s spine health and provides an empirical data basis for policy-making. The multi-dimensional distribution was examined, showing that scoliosis is more common in girls at puberty, with the idiopathic type and mild curvature being the most common, thus providing a basis for targeted and individual interventions. The cross-sectional study was addressed while the ensuing national intervention, treatment, and prevention were neglected, and our data outcomes may highlight the implementation of the following work. Notably, the distribution features of scoliosis could provide insights for its aetiological research.

Several limitations to our study should be noted. First, much data was missing for the third screening outcome, especially data from the northwestern and central regions, even though 2.2 million students were enrolled. Those exacerbated the challenges during our study’s execution and weakened pooled outcomes’ inferential strength. Second, selecting and reporting bias was inevitable because of the low quality and evidence levels and the large heterogeneity in screening for the non-uniform designation derived from the cross-sectional studies [99]. Nevertheless, this first-hand data are irreplaceable and essential for characterising the scoliosis distribution in mainland China. Third, the positive rate of scoliosis was not its actual prevalence since the cases of scoliosis in dropout children were neglected in all studies. That population probably had psychosocial issues caused by the spinal deformity [100], which would merit special attention. Finally, scoliosis prevalence differed among provinces partly due to confounding factors, such as the frequency and quality of their field surveys of school children (e.g. in Shanghai vs. Tibet). Given these caveats, it is imperative to design and carry out nationwide screening of scoliosis in a standardised way (uniform designation and implementation).

## CONCLUSIONS

Based on records for 2.22 million school children and adolescents in mainland China, we identified the prevalence of scoliosis and its distribution characteristics. Its positive rate from primary screening and prevalence was 3.97% and 1.20%, respectively. The prevalence in girls was about 1.57 times higher than in boys, and the peak age group prevalence among girls was one to two years earlier, coinciding with their onset of puberty. The highest prevalence was found in the central region. The most common type of scoliosis was idiopathic deformity, at 1.18%, while mild scoliosis characterised most cases (80%). The high congruence between primary and final screening regarding their spatial distribution suggests that primary screening is repeatable and credible. Collectively, these findings suggest that scoliosis among students in mainland China is a burden on public health. This study could be a key spur for policy-makers and researchers to organise regional and nationwide screening, prevent and control scoliosis through regular school entrance examinations, and promote fundamental research on scoliosis.

## Additional material


Online Supplementary Document

## References

[1] Jada A Mackel CE Hwang SW Samdani AF Stephen JH Bennett JT Evaluation and management of adolescent idiopathic scoliosis: a review. Neurosurg Focus. 2017;43:E2. 10.3171/2017.7.FOCUS17297 28965447

[2] Hsu PC Feng CK Huang SH Chiu JW Chou CL Yang TF Health-related quality of life in children and adolescent with different types of scoliosis: a cross-sectional study. J Chin Med Assoc. 2019;82:161–5. 10.1097/JCMA.0000000000000020 30839509

[3] Campbell M Matsumoto H St HT Roye BD Roye DP Vitale MG Burden of care in families of patients with early onset scoliosis. J Pediatr Orthop B. 2020;29:567–71. 10.1097/BPB.0000000000000711 31895294

[4] Ueno M Takaso M Nakazawa T Imura T Saito W Shintani R A 5-year epidemiological study on the prevalence rate of idiopathic scoliosis in Tokyo: school screening of more than 250,000 children. J Orthop Sci. 2011;16:1–6. 10.1007/s00776-010-0009-z 21293892

[5] Lee JY Moon SH Kim HJ Park MS Suh BK Nam JH The prevalence of idiopathic scoliosis in eleven year-old Korean adolescents: a 3 year epidemiological study. Yonsei Med J. 2014;55:773–8. 10.3349/ymj.2014.55.3.773 24719147 PMC3990085

[6] Hengwei F Zifang H Qifei W Weiqing T Nali D Ping Y Prevalence of idiopathic scoliosis in Chinese schoolchildren: a large, population-based study. Spine. 2016;41:259–64. 10.1097/BRS.0000000000001197 26866739

[7] Zhang H Guo C Tang M Liu S Li J Guo Q Prevalence of scoliosis among primary and middle school students in Mainland China: a systematic review and meta-analysis. Spine. 2015;40:41–9. 10.1097/BRS.0000000000000664 25341979

[8] Yong F Wong HK Chow KY Prevalence of adolescent idiopathic scoliosis among female school children in Singapore. Ann Acad Med Singap. 2009;38:1056–63. 10.47102/annals-acadmedsg.V38N12p1056 20052440

[9] Fong DY Cheung KM Wong YW Wan YY Lee CF Lam TP A population-based cohort study of 394,401 children followed for 10 years exhibits sustained effectiveness of scoliosis screening. Spine J. 2015;15:825–33. 10.1016/j.spinee.2015.01.019 25615844

[10] Zheng Y Dang Y Yang Y Sun N Wang T Li H A case-control study of body composition, prevalence, and curve severity of the patients with adolescent idiopathic scoliosis in the east part of China. Spine Deform. 2017;5:374–80. 10.1016/j.jspd.2017.04.002 29050712

[11] Fong DY Lee CF Cheung KM Cheng JC Ng BK Lam TP A meta-analysis of the clinical effectiveness of school scoliosis screening. Spine. 2010;35:1061–71. 10.1097/BRS.0b013e3181bcc835 20393399

[12] von Elm E Altman DG Egger M Pocock SJ Gotzsche PC Vandenbroucke JP The strengthening the reporting of observational studies in epidemiology (STROBE) statement: guidelines for reporting observational studies. Int J Surg. 2014;12:1495–9. 10.1016/j.ijsu.2014.07.013 25046131

[13] Qiu Y [Epidemiological investigation of scoliosis among primary and secondary school students in Jiangyin City]. Experience Exch. 2022;7:90-2. Chinese.

[14] Wen X Xu H Liu N Qian L Huang C [Analysis of abnormal spinal curvature results of primary and secondary school students aged 7~18 in Mianyang City in 2019]. J Prev Med Inf. 2021;37:828–32. Chinese.

[15] Li Z Zhou J Zhou Y Chen Y Zhou J [Epidemiological investigation of scoliosis in adolescents and children in an art training school in Leshan City]. Westleather. 2021;43:50–2. Chinese.

[16] Chen Y Li L Yang H Hu W Jia F Zhai F [Current status and influencing factors of scoliosis of children in Shijiazhuang]. Chin J Sch Health. 2021;42:1674–8. Chinese.

[17] Sun Y Liu J Xiong L Li M Chen S He W [Scoliosis and associated factors among secondary school students in Guangzhou City]. Chin J Sch Health. 2021;42:1867–73. Chinese.

[18] Cai Z Wu R Zheng S Qiu Z Wu K [Morphology and epidemiological study of idiopathic scoliosis among primary school students in Chaozhou, China]. Environ Health Prev Med. 2021;26:71–80. Chinese. 10.1186/s12199-021-00989-3 34217201 PMC8254979

[19] Ding X Teng J Chai S Li X Su X Tong S [A survey of prevalence rate of idiopathic scoliosis of secondary school students in Zhengdong new district of Zhengzhou]. J Traditl Chin. Orthop Traumatol. 2020;32:31–4. Chinese.

[20] Yang L Lu X Yan B Huang Y Prevalence of incorrect posture among children and adolescents: finding from a large population-based study in China. iScience. 2020;23:101043. 10.1016/j.isci.2020.101043 32330860 PMC7178490

[21] Xia C Guan J Ma L Cai Y Shen J [Investigation of prevalence of scoliosis among adolescents in a community of Jiading District, Shanghai]. Shanghai Med Pharm J. 2019;40:53–5. Chinese.

[22] Zeng L [Investigation on the prevalence of adolescent scoliosis in Huizhou area]. Shenzhen J Integrated Tradl Chin West Med. 2019;29:196–7. Chinese.

[23] Wang Y Chen X Yuan X Cui L Wang Y Liu Y [The epidemiological investigation of adolescent scoliosis in Beijing Tongzhou district]. Zhongguo Jizhu Jisui Zazhi. 2018;28:667–9. Chinese.

[24] Wei C Lu Z Huang X Sun L Long X Zeng Y [Screening for abnormal angle of trunk rotation among adolescents in Yichang city]. Chin J Gen Pract (Los Angel). 2018;17:59–62. Chinese.

[25] Li M Su L Zhong H Zeng L Huang Q Kang J [An investigation about the prevalence rate of idiopathic scoliosis in secondary school students in Shenzhen]. Shenzhen J Integrated Traditl Chin West Med. 2018;28:3–5. Chinese.

[26] Du J Cai S Jiang B Zhao Z Ma Z [Survey analysis of idiopathic scoliosis in 12881 secondary school students from Shantou city, Guangdong Province]. Zhongguo Jiceng Yiyao. 2018;25:1976–9. Chinese.

[27] Huang F Wu J Huang S Zhang Z Cao H Zeng Z [Investigation and analysis of the prevalence of idiopathic scoliosis among secondary school students in Zhongshan City, Guangdong Province]. J Front Med. 2018;8:374–5. Chinese.

[28] Deng X Wu Y Deng M [Investigation on current situation of adolescent scoliosis in Ganzi Tibetan Autonomous Prefecture in 2018]. J Prev Med Inf. 2019;35:667–70. Chinese.

[29] Wang H Sun Z Wang T Duan Y [Prevalence and risk factors of adolescent idiopathic scoliosis in Kunming]. Chin J Sch Health. 2018;39:1851–4. Chinese.

[30] He Y Guan B Wang X Zhu B Wu S, A H [Investigation on the incidence of adolescent idiopathic scoliosis of junior high school students in Xining City]. Qinghai Med J (Ft Sam Houst, Tex). 2018;48:69–71. Chinese.

[31] Tang Q Zhu M Shang Y Zhang J Yu X Guo Y [Investigation on the prevalence of idiopathic scoliosis among junior high school students in the former Jing'an District of Shanghai]. Int J Orthop (Hong Kong). 2017;38:205–6. Chinese.

[32] Miao G Xu C [Epidemiological survey of scoliosis among adolescents in Jiangyin City]. Jiangsu J Prev Med (Wilmington). 2017;28:195-213. Chinese.

[33] Nie Y Jin Z Zhang L Zhang L Jin W Ying X [Network screening and early intervention of adolescent idiopathic scoliosis]. China Mod Doct. 2017;55:16–20. Chinese.

[34] Li Y Cui W Yan X Wang H [Epidemiology of congenital scoliosis in Luohe]. Zhonghua Xiaoerwaike Zazhi. 2017;38:221–4. Chinese.

[35] Deng W Zhang J Du R Wang X [Abnormal spinal curvature situation and influencing factors in school children in Xiangyang City]. Chin J Sch Doct. 2016;30:285–7. Chinese.

[36] Hu G Liu C Liu H Yan B Li X Yang D [Prevalence and prevention of idiopathic scoliosis among secondary school students in Nanshan district of Shenzhen city]. Chin Community Doct. 2017;33:110–1. Chinese.

[37] Han K Guo T Liu H Qu Q [Incidence and imaging characteristics of idiopathic scoliosis among college entrance examination students in Qilihe District and Anning District, Lanzhou City in 2016]. J Imag Res Appl. 2017;1:85–6. Chinese.

[38] Du Q Zhou X Negrini S Chen N Yang X Liang J Scoliosis epidemiology is not similar all over the world: a study from a scoliosis school screening on Chongming Island (China). BMC Musculoskelet Disord. 2016;17:303–10. 10.1186/s12891-016-1140-6 27444153 PMC4957389

[39] Zheng Y Wu X Dang Y Yang Y Reinhardt JD Dang Y Prevalence and determinants of idiopathic scoliosis in primary school children in Beitang district, Wuxi, China. J Rehabil Med. 2016;48:547–53. 10.2340/16501977-2098 27166625

[40] He T Zhang J [Analysis the scoliosis in Jinjiang primary and secondary school students]. J Prim Med Forum. 2016;20:5081–2. Chinese.

[41] Huang Z Chen L Zhang Y Shui L Cui L [Analysis of the detection results of scoliosis in primary and secondary school students in Kunming]. Hainan Med J (Ft Sam Houst, Tex). 2016;27:2390–1. Chinese.

[42] Chen J Yang F Guo H Hao D Zhou J Luo C [Investigation and research on scoliosis of urban adolescents in Xi’an city]. Shaanxi Med J (Ft Sam Houst, Tex). 2016;45:371–3. Chinese.

[43] Ke R Cao X Huang Y Liu F Chen Y Li F [Prevalence of adolescent idiopathic scoliosis in Zhenjiang]. Jiangsu Med J (Ft Sam Houst, Tex). 2015;41:2130–2. Chinese.

[44] Ma J Liu Q Chen Z Lin M Gao Z [Epidemiology of adolescent idiopathic scoliosis in Sanya]. Hainan Med J (Ft Sam Houst, Tex). 2015;26:2000–2. Chinese.

[45] Chen J Wang J Yan X Meng S Feng H Wang H [Epidemiological investigation of scoliosis in primary and secondary school students in Xi’an]. J Trad Chin Orthop Trauma. 2015;27:17–20. Chinese.

[46] Yu S Hu H Fan Z Qiu Q Li Y [An investigation on the prevalence rate of idiopathic scoliosis for primary and secondary school students in Guangzhou]. Clin Mediterr Eng. 2014;21:1359–60. Chinese.

[47] Zhao Z Lan H Wang Z Feng J Wu D [Investigation and study of prevalence rate of adolescent scoliosis in Guangzhou Liwan district]. Chin Mod Med. 2014;21:137–9. Chinese.

[48] Ren K Gong X Zhang R Zeng X Zhan J Liu J [Investigation of AIS among school children in Zigong]. Sichuan Med J (Ft Sam Houst, Tex). 2014;35:853–5. Chinese.

[49] Wang Y Wu B Lin Y [A scoliosis-prevalence survey of students in primary and secondary schools in Wenzhou city]. J Trad Chin Orthop Trauma. 2013;25:25–7. Chinese.

[50] Ke Y He J Pan Z [Investigation on the prevalence of adolescent scoliosis in Foshan City]. J Pract Med (Barc). 2012;28:832–4. Chinese.

[51] Chen L Chen H Lin J Ao R Lai H Zeng X [A general survey of scoliosis in primary and secondary school students in YangJiang Area of Guangdong Province]. Med Innov Chin. 2012;18:89–91. Chinese. 10.1186/s13020-023-00782-0

[52] Zhang S Ma S Liu H Li Z Wang X Ren X [The study about the growing development and scoliosis of Mongolian, Han and Hui pupils in Inner Mongolia]. J Dis Monit Control. 2011;5:131–3. Chinese.

[53] Liu W Chen Q Wang L [A general survey of the adolescent scoliosis in Harbin and an analysis of the result of the brace treatment]. Ortho J China. 2011;19:1244–7. Chinese.

[54] Huang NQ Guo HS Liu J Huang GX Yang XH Chen J [A survey on adolescent scoliosis in Guangzhou]. Zhonghua Liu Xing Bing Xue Za Zhi. 2011;32:138–41. Chinese. 21518621

[55] Tang S Fu G [Screening for spinal deformities in 40579 students]. Linchuang Xiaoer Waike Zazhi. 2011;10:430–3. Chinese.

[56] Li Q Yuan Y Chen D Zhang A Mei Z Zhao C [A survey on adolescent scoliosis under the step intervention]. J Clin Orthod. 2011;14:481–3. Chinese.

[57] Chen C Tong B Cong Y Wang Y He Z Chen Z [Investigation of scoliosis among school children in Jinzhou of Liaoning]. Med Innov China. 2010;7:44–6. Chinese.

[58] Lu M Chen Q Gao J Zhang C Qu J [A general survey of adolescent scoliosis in Heilongjiang province]. Orthop J China. 2010;18:591–3. Chinese.

[59] Yu H Liu Z Zou A Zhang L Wei W Wang Y [Analysis of the current situation and influencing factors of scoliosis in primary and secondary school students in Xiamen]. Chin J Sch Health. 2010;31:1271–2. Chinese.

[60] Du Q Yin H Huang M Zeng Q Zhao H Lin Y [A survey on the incidence of idiopathic scoliosis for primary and secondary school students in Shunde District]. Lingnan Mod. Clin Surg. 2010;10:52–4. Chinese.

[61] Dong Z Xiong L Zhang J Tang X Xiao Q [Investigation of scoliosis among school children in Nanchang]. Acta Acad Med Jiangxi. 2009;49:129–32. Chinese.

[62] Zhou H Zhang J Lin S [Epidemiological investigation of scoliosis among adolescents in Hui′ an county, Fujian Province]. Zhongguo Jizhu Jisui Zazhi. 2008;18:824–7. Chinese.

[63] Zhang J Lin G Zeng X Gao T Liu X [A Survey on the incidence of juvenile scoliosis in Quanzhou Area]. Chin J Trad Med Traum Orthop. 2008;16:1–4. Chinese.

[64] Sun R Yin X Liu G Wen C Zhang X Liu Y [Analysis of the results of the prevalence survey of adolescent scoliosis in the urban area of Liupanshui, Guizhou Province in 2007]. Guizhou Med J (Ft Sam Houst, Tex). 2009;33:73–4. Chinese.

[65] Wang Z Li Z Liu Z Wang W Chen Y Zhao J [Investigation of scoliosis among school children in Beijing]. Zhongguo Jizhu Jisui Zazhi. 2007;17:440–2. Chinese.

[66] Yu H Sun B Zhan Q [The relationship between adolescent scoliosis and habits and diet]. Zhejiang Trauma Surg J (NY). 2006;11:203–4. Chinese.

[67] Cheng B Li F Song J [A general survey and treatment of children and adolescent scoliosis in Xi′an]. Zhongguo Jizhu Jisui Zazhi. 2006;16:180–2. Chinese.

[68] Liang X Huang S Yu B Chen Z [General survey and prevention of scoliosis in young children in Zhaoqing City, Guangdong Province]. Zhongguo Fuyou Baojian. 2005;20:1496–7. Chinese.

[69] Gao W Ni X Shao Y [The application and revelation of “point Line” in scoliosis investigation]. Hebei Med. 2004;10:1073–5. Chinese.

[70] Meng L Meng L Ao B Wang Z [An epidemiological survey of scoliosis among primary and junior secondary school students in Langfang Area]. J Med Theor Prac. 2003;16:516–8. Chinese.

[71] Zhang S Xing S Jin X Liang N Lin Y Huang S [A survey on scoliosis in school-age population in Hainan and melatonin levels in idiopathic scoliosis]. Orthop J Chin. 2003;11:1712–4. Chinese.

[72] Liu S Li W Li Y [A survey on adolescent scoliosis in Guangdong Province]. Zhongguo Jizhu Jisui Zazhi. 2002;12:41–3. Chinese.

[73] Liang D Ye D Yao W Qiu S Li T Luo D [Investigation on the chest health of adolescent students in Lhasa]. Tibetan J Med. 2002;23:5–6. Chinese.

[74] Li W Liu S Chen Z [A survey on scoliosis in school-age population in Guangzhou]. Zhonghua Xiaoerwaike Zazhi. 2001;22:104–6. Chinese.

[75] Li Y Li Z Huang S Xu R He H Liu G [General survey and early treatment of adolescent scoliosis in mountainous areas of northern Guangdong]. Chin J Sch Doct. 1999;13:414–5. Chinese.

[76] Li Q Liu S Xu Z Li W Lu Z Cai Q [A general survey and treatment of scoliosis in primary and secondary school students in Zhongshan of Guangdong]. Zhonghua Guke Zazhi. 1999;19:265–8. Chinese.

[77] Wang X Wang S [An analysis of 13 560 adolescent scoliosis screening]. New Med (Wars). 1998;29:534–5. Chinese.

[78] Wang YP Ye Q Wu B Wu Z [Result on the screening of scoliosis among school students in Beijing area]. Chin Zhonghua Liu Xing Bing Xue Za Zhi. 1996;17:160–2. Chinese. 9208515

[79] Zhao G Tian J Wu X Shi Q [Investigation on the prevalence of scoliosis among students in some primary and secondary schools in Shanghai]. Chin J Traditl Med Traumatol Orthop. 1996;4:28–9. Chinese.

[80] Yu Z Qu Z Wang M Xiao L Cheng G Zhang S [Middle and primary school children screening for scoliosis in rural areas- early diagnosis and treatment]. Zhonghua Guke Zazhi. 1995;15:418–20. Chinese.

[81] Ma X Zhao B Lin Q Wang G Li H [Investigation on scoliosis incidence among 24130 school children]. Zhonghua Liu Xing Bing Xue Za Zhi. 1995;16:109–10. Chinese. 7781047

[82] Jiang H Jiang Y Zhao C Tian C Wang J Li L [Mass survey of school children in Tianjin for scoliosis]. Zhonghua Guke Zazhi. 1994;14:362–4. Chinese.

[83] Chen B Zhou B Chen X Zou Y. [Trunk asymmetry and primary scoliosis-an investigation of scoliosis in secondary school students in Qingdao]. J Cervicodynia Lumbodynia. 1990;11:1–4. Chinese.

[84] Cao P Liu Y Li S [Health status of Korean primary and secondary school students]. Chin J Sch Doct. 1989;3:21–3. Chinese.

[85] Zhang GP Li ZR Wei XR Cao YL Cui QL Screening for scoliosis among school children in Beijing. Chin Med J (Engl). 1988;101:151–4. 3136984

[86] Tan C Lu B Ruan Q Chen S Mo Y Zhang W [Investigation report on poor eyesight trachoma, dental caries and scoliosis of 33079 primary and secondary school students in Guangxi in 1987]. Guangxi Med J (Ft Sam Houst, Tex). 1988;10:314–5. Chinese.

[87] Wang X Xiao L Xu J Qian J Ouyang R Gu C [Investigation and analysis of spine curvature of primary and secondary school students in Nanjing]. J Nanjing Med Univ. 1985;5:209–11. Chinese.

[88] Pin LH Mo LY Lin L Hua LK Hui HP Hui DS Early diagnosis of scoliosis based on school-screening. J Bone Joint Surg Am. 1985;67:1202–5. 10.2106/00004623-198567080-00009 4055844

[89] Płaszewski M Grantham W Jespersen E Screening for scoliosis-new recommendations, old dilemmas, no straight solutions. World J Orthop. 2020;11:364–79. 10.5312/wjo.v11.i9.364 32999857 PMC7507078

[90] Suh SW Modi HN Yang JH Hong JY Idiopathic scoliosis in Korean schoolchildren: a prospective screening study of over 1 million children. Eur Spine J. 2011;20:1087–94. 10.1007/s00586-011-1695-8 21274729 PMC3176687

[91] Jin J Screening for scoliosis in adolescents. JAMA. 2018;319:202. 10.1001/jama.2017.20372 29318279

[92] Li C Miao J Gao X Zheng L Su X Hui H Factors associated with caregiver burden in primary caregivers of patients with adolescent scoliosis: a descriptive cross-sectional study. Med Sci Monit. 2018;24:6472–9. 10.12659/MSM.909599 30218532 PMC6151967

[93] Zale CL McIntosh AL Adolescent idiopathic scoliosis for pediatric providers. Pediatr Ann. 2022;51:e364–9. 10.3928/19382359-20220724-01 36098613

[94] Chung LY Nam HK Rhie Y Huh R Lee KH Prevalence of idiopathic scoliosis in girls with central precocious puberty: effect of a gonadotropin-releasing hormone agonist. Ann Pediatr Endocrinol Metab. 2020;25:92–6. 10.6065/apem.1938164.082 32615688 PMC7336263

[95] Wise CA What causes AIS? Ask the genome! Stud Health Technol Inform. 2021;280:3–8. 34190051 10.3233/SHTI210423

[96] Grivas TB Koukos K Koukou UI Maziotou C Polyzois BD The incidence of idiopathic scoliosis in Greece–analyais of domestic school screening programs. Stud Health Technol Inform. 2002;91:71–5. 15457697

[97] Applebaum A Ference R Cho W Evaluating the role of surface topography in the surveillance of scoliosis. Spine Deform. 2020;8:397–404. 10.1007/s43390-019-00001-7 31965557

[98] Wong AYL Chan TPM Chau AWM Tung CH Kwan KCK Lam AKH Do different sitting postures affect spinal biomechanics of asymptomatic individuals? Gait Posture. 2019;67:230–5. 10.1016/j.gaitpost.2018.10.028 30380507

[99] Guyatt GH Oxman AD Vist GE Kunz R Falck-Ytter Y Alonso-Coello P GRADE: an emerging consensus on rating quality of evidence and strength of recommendations. BMJ. 2008;336:924–6. 10.1136/bmj.39489.470347.AD 18436948 PMC2335261

[100] Gallant JN Morgan CD Stoklosa JB Gannon SR Shannon CN Bonfield CM Psychosocial difficulties in adolescent idiopathic scoliosis: body image, eating behaviors, and mood disorders. World Neurosurg. 2018;116:421–432.e1. 10.1016/j.wneu.2018.05.104 29803063

