# Supplementary material for: Distribution of scoliosis in 2.22 million adolescents in mainland China: A population-wide analysis
Source: J Glob Health. 2024 Jul 19;14:04117. doi: 10.7189/jogh.14.04117 (PMC11258535; doi:10.7189/jogh.14.04117)
Supplement: Online Supplementary Document [file jogh-14-04117-s001.pdf]

Study or Subgroup	Girl Events	Girl Total	Boy Events	Boy Total	Weight	Odds Ratio M-H, Random, 95% CI
**1.2.1 western**						
Guizhou Liupanshui2008	1094	8090	800	9465	3.9%	1.69 [1.54, 1.87]
Shaanxi Xi'an2006	687	11850	702	13875	3.9%	1.15 [1.04, 1.29]
Sichuan Mianyang2021	405	3991	322	4185	3.8%	1.35 [1.16, 1.58]
Sichuan Zigong2014	307	7591	264	9757	3.8%	1.52 [1.28, 1.79]
Yunnan Kunming2018	36	469	14	315	2.5%	1.79 [0.95, 3.37]
**Subtotal (95% CI)**		**31991**		**37597**	**18.0%**	**1.44 [1.20, 1.72]**
Total events	2529		2102			
Heterogeneity: $Tau^2 = 0.03$; $Chi^2 = 28.24$, df = 4 (P < 0.0001); $I^2 = 86\%$						
Test for overall effect: Z = 3.88 (P = 0.0001)						
**1.2.2 middle**						
Heilongjiang Harbin2011	639	12140	601	12222	3.9%	1.07 [0.96, 1.20]
Heilongjiang2010	463	8508	448	9017	3.9%	1.10 [0.96, 1.26]
Hubei Xiangyang2017	41	943	60	1111	3.2%	0.80 [0.53, 1.20]
Inner Mongolia Huhhot2011	32	630	23	630	2.7%	1.41 [0.82, 2.44]
Shanxi Changzhi2005	917	11583	878	12547	3.9%	1.14 [1.04, 1.26]
**Subtotal (95% CI)**		**33804**		**35527**	**17.6%**	**1.11 [1.04, 1.18]**
Total events	2092		2010			
Heterogeneity: $Tau^2 = 0.00$; $Chi^2 = 3.96$, df = 4 (P = 0.41); $I^2 = 0\%$						
Test for overall effect: Z = 3.11 (P = 0.002)						
**1.2.3 eastern**						
Beijing1988	668	10135	554	10283	3.9%	1.24 [1.10, 1.39]
Guangdong Chaozhou2021	216	2479	122	3018	3.7%	2.27 [1.80, 2.85]
Guangdong Guangzhou2021	97	1021	77	1100	3.5%	1.39 [1.02, 1.91]
Guangdong Huizhou2019	1842	45546	1584	58542	4.0%	1.52 [1.42, 1.62]
Guangdong Shantou2018	663	6255	880	6626	3.9%	0.77 [0.70, 0.86]
Guangdong Shenzhen2020	15398	270125	8774	324932	4.0%	2.18 [2.12, 2.24]
Guangdong2016	5627	49111	2285	50584	4.0%	2.74 [2.60, 2.88]
Guangxi Nanning1987	143	14881	133	18198	3.7%	1.32 [1.04, 1.67]
Hainan Sanya2015	184	3202	191	3750	3.7%	1.14 [0.92, 1.40]
Jiangsu Nanjing1985	209	1231	135	1336	3.7%	1.82 [1.44, 2.29]
Jiangsu Wuxi2016	92	5116	190	5908	3.6%	0.55 [0.43, 0.71]
Shandong Qingdao1990	624	10246	918	11187	3.9%	0.73 [0.65, 0.81]
Shanghai1996	365	4843	198	5230	3.8%	2.07 [1.73, 2.47]
Shanghai2016	241	3347	201	3477	3.8%	1.26 [1.04, 1.53]
Shanghai2017	394	2579	126	2748	3.7%	3.75 [3.05, 4.62]
Tianjin1994	369	18154	299	18849	3.8%	1.29 [1.10, 1.50]
Zhejiang Hangzhou2006	204	3467	127	3671	3.7%	1.74 [1.39, 2.19]
**Subtotal (95% CI)**		**451738**		**529439**	**64.4%**	**1.47 [1.19, 1.81]**
Total events	27336		16794			
Heterogeneity: $Tau^2 = 0.19$; $Chi^2 = 1198.33$, df = 16 (P < 0.00001); $I^2 = 99\%$						
Test for overall effect: Z = 3.55 (P = 0.0004)						
**Total (95% CI)**		**517533**		**602563**	**100.0%**	**1.39 [1.18, 1.64]**
Total events	31957		20906			
Heterogeneity: $Tau^2 = 0.17$; $Chi^2 = 1562.28$, df = 26 (P < 0.00001); $I^2 = 98\%$						
Test for overall effect: Z = 3.95 (P < 0.0001)						
Test for subgroup differences: $Chi^2 = 12.21$, df = 2 (P = 0.002), $I^2 = 83.6\%$						

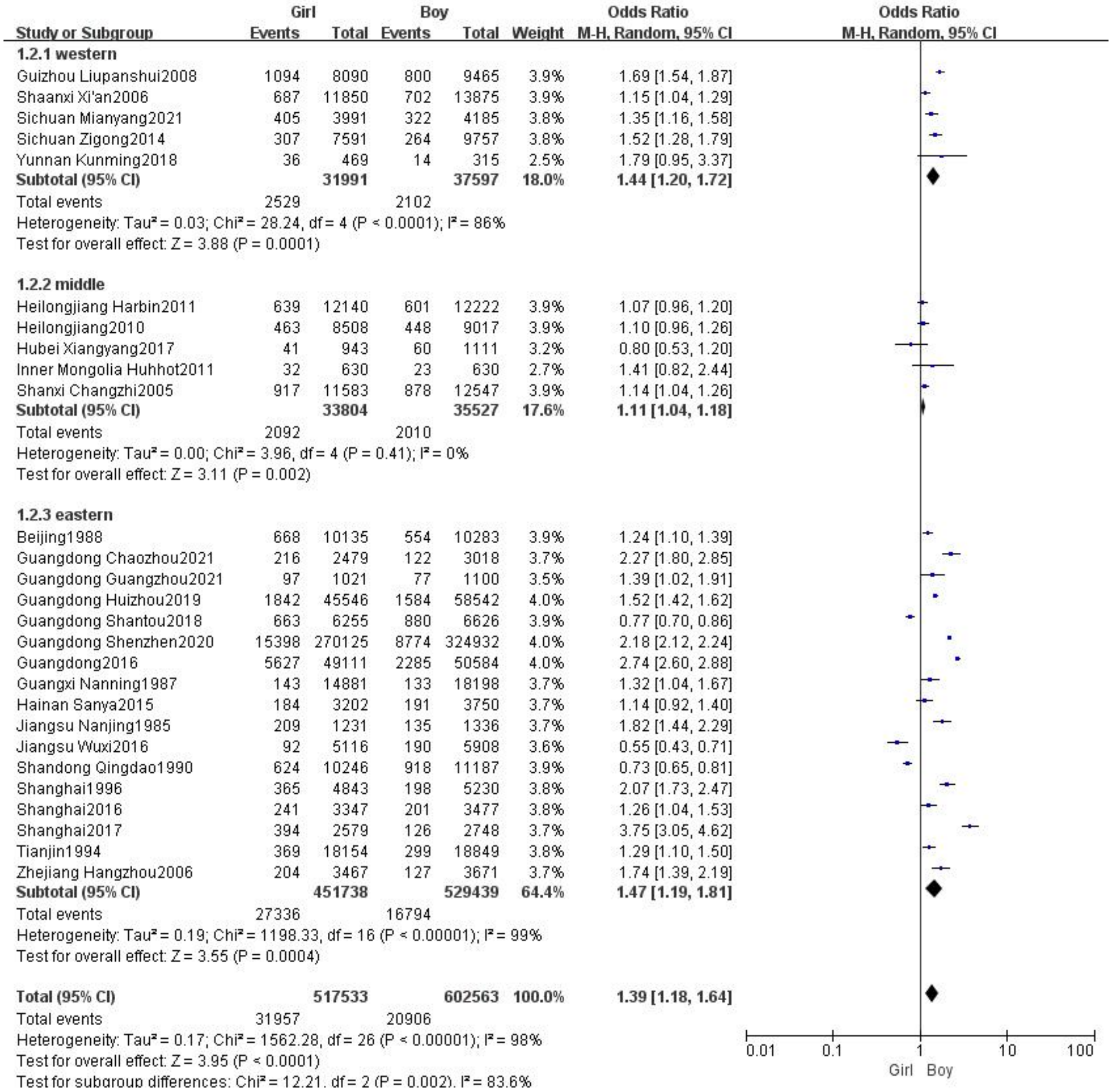

Figure S1 Panel A

Study or Subgroup	Girl Events	Girl Total	Boy Events	Boy Total	Weight	Odds Ratio M-H, Random, 95% CI
**2.3.1 western**						
Gansu Lanzhou2017	38	2985	14	2395	1.6%	2.19 [1.19, 4.06]
Guizhou Liupanshui2008	109	8090	75	9465	2.2%	1.71 [1.27, 2.30]
Shaanxi Xi'an2006	179	11850	164	13875	2.3%	1.28 [1.04, 1.59]
Shaanxi Xi'an2015	233	14844	160	15898	2.3%	1.57 [1.28, 1.92]
Shaanxi Xi'an2016	214	13081	146	14809	2.3%	1.67 [1.35, 2.06]
Sichuan Ganzi2018	69	2381	54	2745	2.1%	1.49 [1.04, 2.13]
Sichuan Zigong2014	48	7591	41	9757	2.0%	1.51 [0.99, 2.29]
Tibet Lhasa2002	10	2990	9	2747	1.2%	1.02 [0.41, 2.52]
**Subtotal (95% CI)**		**63812**		**71691**	**16.1%**	**1.53 [1.38, 1.70]**
Total events	900		663			
Heterogeneity: Tau² = 0.00; Chi² = 6.01, df = 7 (P = 0.54); I² = 0%						
Test for overall effect: Z = 8.25 (P < 0.00001)						
**2.3.2 middle**						
Heilongjiang Harbin2011	227	12140	196	12222	2.3%	1.17 [0.96, 1.42]
Heilongjiang2010	188	8508	147	9017	2.3%	1.36 [1.10, 1.70]
Henan Luohe2017	21	7095	8	7231	1.3%	2.68 [1.19, 6.06]
Henan Zhengzhou2020	149	9201	58	9317	2.2%	2.63 [1.94, 3.56]
Hunan Changsha1985	97	3963	74	4202	2.2%	1.40 [1.03, 1.90]
Inner Mongolia Huhhot2011	7	32	3	23	0.6%	1.87 [0.43, 8.16]
Jiangxi Nanchang2009	41	4675	23	5444	1.8%	2.09 [1.25, 3.48]
Shanxi Changzhi2005	187	11583	160	12547	2.3%	1.27 [1.03, 1.57]
**Subtotal (95% CI)**		**57197**		**60003**	**15.1%**	**1.58 [1.27, 1.97]**
Total events	917		669			
Heterogeneity: Tau² = 0.06; Chi² = 25.43, df = 7 (P = 0.0006); I² = 72%						
Test for overall effect: Z = 4.04 (P < 0.0001)						
**2.3.3 eastern**						
Beijing1988	127	10135	86	10283	2.2%	1.50 [1.14, 1.98]
Beijing2018	41	12165	40	12932	2.0%	1.09 [0.70, 1.69]
Fujian Quanzhou2008A	141	15068	93	17212	2.3%	1.74 [1.34, 2.26]
Fujian Quanzhou2008B	80	9776	78	11336	2.2%	1.19 [0.87, 1.63]
Fujian Quanzhou2016	85	9924	69	11491	2.2%	1.43 [1.04, 1.97]
Guangdong Foshan2012	89	9154	67	9644	2.2%	1.40 [1.02, 1.93]
Guangdong Guangzhou2001	203	16154	158	17644	2.3%	1.41 [1.14, 1.74]
Guangdong Guangzhou2011	180	14758	31	15384	2.1%	6.12 [4.17, 8.96]
Guangdong Guangzhou2014A	141	17195	109	12337	2.3%	0.93 [0.72, 1.19]
Guangdong Guangzhou2014B	54	4140	31	4211	1.9%	1.78 [1.14, 2.78]
Guangdong Huizhou2019	288	45546	246	58542	2.4%	1.51 [1.27, 1.79]
Guangdong Shaoguan1999	45	10462	29	11397	1.9%	1.69 [1.06, 2.70]
Guangdong Shenzhen2018	71	7801	55	7446	2.1%	1.23 [0.87, 1.76]
Guangdong Yangjiang2012	70	8985	66	10661	2.1%	1.26 [0.90, 1.77]
Guangdong Zhaoqing2005	33	4051	39	4159	1.9%	0.87 [0.54, 1.38]
Guangdong Zhongshan1999	53	8213	59	10116	2.1%	1.11 [0.76, 1.61]
Guangdong Zhongshan2018	769	19916	649	21432	2.4%	1.29 [1.16, 1.43]
Guangdong Zhuhai1998	57	6491	47	7069	2.0%	1.32 [0.90, 1.95]
Guangdong2002	366	40594	287	46952	2.4%	1.48 [1.27, 1.73]
Guangdong2016	3870	49111	1255	50584	2.5%	3.36 [3.15, 3.59]
Hainan Haikou2003	8	3775	9	4423	1.1%	1.04 [0.40, 2.70]
Hainan Sanya2015	46	3202	42	3750	2.0%	1.29 [0.84, 1.96]
Hebei Langfang2003	141	8157	110	8501	2.3%	1.34 [1.04, 1.72]
Jiangsu Nantong2004	31	4257	34	4395	1.9%	0.94 [0.58, 1.53]
Jiangsu Wuxi2017	129	30434	71	36888	2.2%	2.21 [1.65, 2.95]
Jiangsu Wuxi2022	97	9427	66	9135	2.2%	1.43 [1.04, 1.96]
Jiangsu Zhenjiang2015	120	7723	41	7944	2.1%	3.04 [2.13, 4.34]
Liaoning Jinzhou2010	34	5933	7	6324	1.3%	5.20 [2.30, 11.74]
Shanghai1996	280	4843	207	5230	2.4%	1.49 [1.24, 1.79]
Shanghai2016	104	3347	68	3477	2.2%	1.61 [1.18, 2.19]
Shanghai2019	22	1836	11	2077	1.4%	2.28 [1.10, 4.71]
Tianjin1994	242	18154	180	18849	2.3%	1.40 [1.15, 1.70]
Zhejiang Wenzhou2013	63	8835	49	9319	2.1%	1.36 [0.93, 1.98]
**Subtotal (95% CI)**		**429562**		**471144**	**68.8%**	**1.56 [1.30, 1.86]**
Total events	8080		4389			
Heterogeneity: Tau² = 0.23; Chi² = 559.21, df = 32 (P < 0.00001); I² = 94%						
Test for overall effect: Z = 4.88 (P < 0.00001)						
**Total (95% CI)**		**550571**		**602838**	**100.0%**	**1.57 [1.37, 1.79]**
Total events	9897		5721			
Heterogeneity: Tau² = 0.19; Chi² = 633.00, df = 48 (P < 0.00001); I² = 92%						
Test for overall effect: Z = 6.53 (P < 0.00001)						
Test for subgroup differences: Chi² = 0.07, df = 2 (P = 0.96), I² = 0%						

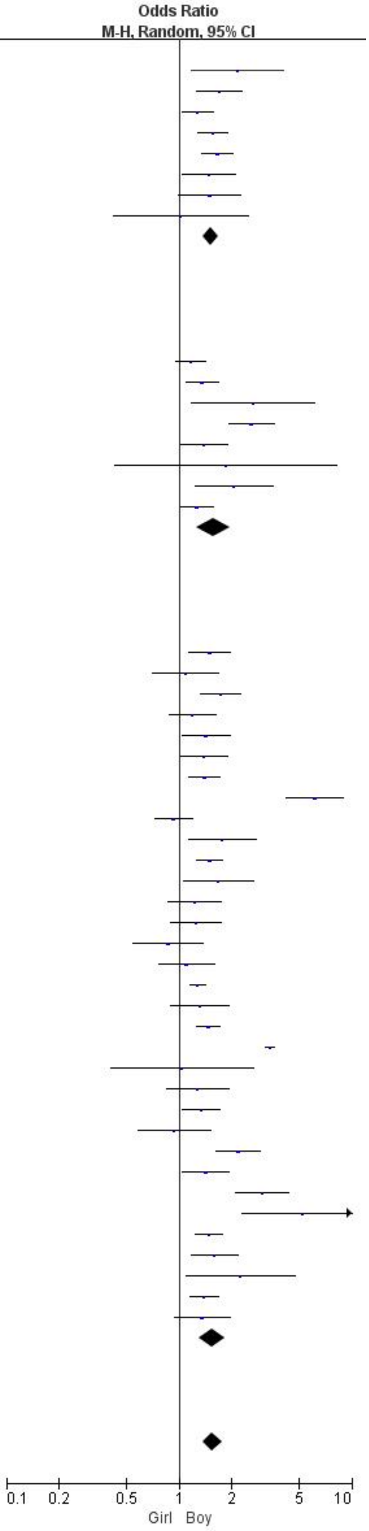

Figure S1 Panel B

Figure S1. Forest plots for the subgroup analysis of scoliosis prevalence by gender and region: **Panel A.** Forest plot of the positive rate from primary screening. **Panel B.** Forest plot of the scoliosis prevalence from final screening.

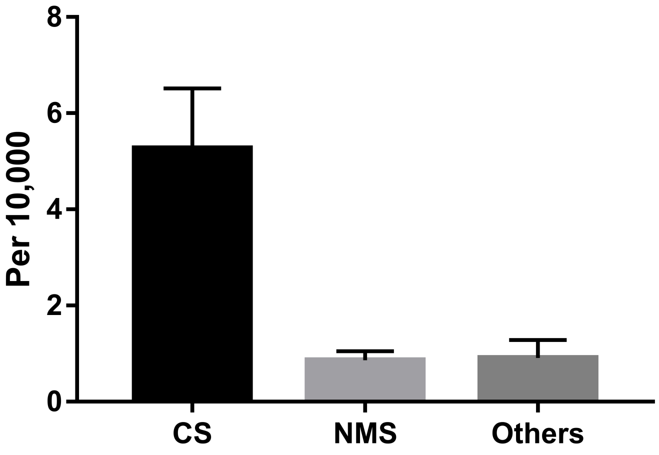

Figure S2. Incidence of three types of scoliosis: CS, NMS, and others pooled. (CS: congenital scoliosis; NMS: neuromuscular scoliosis)

Table S1 Search strategies with databases about the mainland of China

	Searches	Items found
	**PubMed**	
#4	(((((prevalence[Title/Abstract]) OR incidence[Title/Abstract]) OR epidemiology[Title/Abstract]) OR school screening[Title/Abstract])) AND (Search (China[Title/Abstract]) OR China[Affiliation])) AND (Search (scoliosis[Title/Abstract]) OR scolio*[Title/Abstract])	333
#3	China[Title/Abstract]) OR China[Affiliation]	1844671
#2	((prevalence[Title/Abstract]) OR incidence[Title/Abstract]) OR epidemiology[Title/Abstract]) OR school screening[Title/Abstract]	1499787
#1	scoliosis[Title/Abstract]) OR scolio*[Title/Abstract]	22385
	**Scopus**	
#4	TITLE-ABS-KEY (scoliosis OR scolio*) AND (TITLE-ABS-KEY (prevalence OR incidence OR epidemiology OR school OR screening)) AND (TITLE-ABS-KEY(China) OR AFFIL(China))	788
#3	TITLE-ABS-KEY(China) OR AFFIL(China)	7857780
#2	TITLE-ABS-KEY (prevalence OR incidence OR epidemiology OR school OR screening)	4620935
#1	TITLE-ABS-KEY (scoliosis OR scolio*)	36295
	**China National Knowledge Infrastructure (CNKI)**	
#3	(AB=脊柱侧弯 OR AB=脊柱侧凸 OR AB=脊柱畸形 OR AB=scoliosis) AND (AB=流行病学 OR AB=发病率 OR AB=患病率 OR AB=报告 OR AB=调查 OR AB=普查 OR AB=incidence OR AB=prevalence OR AB=epidemiology OR AB=筛查)	1585
#2	AB=流行病学 OR AB=发病率 OR AB=患病率 OR AB=报告 OR AB=调查 OR AB=普查 OR AB=incidence OR AB=prevalence OR AB=epidemiology OR AB=筛查	3335915
#1	AB=脊柱侧弯 OR AB=脊柱侧凸 OR AB=脊柱畸形 OR AB=scoliosis	7734
	**Wan Fang**	
#3	(Abstract=脊柱侧弯 OR Abstract=脊柱侧凸 OR Abstract=脊柱畸形 OR Abstract=scoliosis) AND (Abstract=流行病学 OR Abstract=发病率 OR Abstract=患病率 OR Abstract=报告 OR Abstract=调查 OR Abstract=普查 OR Abstract=incidence OR Abstract=prevalence OR Abstract=epidemiology OR Abstract=筛查)	1880
#2	Abstract=流行病学 OR Abstract=发病率 OR Abstract=患病率 OR Abstract=报告 OR Abstract=调查 OR Abstract=普查 OR Abstract=incidence OR Abstract=prevalence OR Abstract=epidemiology OR Abstract=筛查	1311115
#1	Abstract=脊柱侧弯 OR Abstract=脊柱侧凸 OR Abstract=脊柱畸形 OR Abstract=scoliosis	10487
	**Wei Pu Database**	

#3	(R=脊柱侧弯 + R=脊柱侧凸 + R=脊柱畸形) AND (R=流行病学 + R=发病率 + R=患病率 + R=报告 + R=调查 + R=普查 + R=incidence + R=prevalence + R=epidemiology + R=筛查)	2003
#2	R=流行病学 + R=发病率 + R=患病率 + R=报告 + R=调查 + R=普查 + R=incidence + R=prevalence + R=epidemiology + R=筛查	3880045
#1	R=脊柱侧弯 + R=脊柱侧凸 + R=脊柱畸形	10188
	**China National Science and Technology Digital Library (NSTL)**	
#3	((((脊柱侧凸) or 脊柱侧弯) or 脊柱畸形) or scoliosis) and (((((((((流行病学) or 发病率) or 患病率) or 报告) or 调查) or 普查) or incidence) or prevalence) or epidemiology) or 筛查)	290
#2	(((((((((流行病学) or 发病率) or 患病率) or 报告) or 调查) or 普查) or incidence) or prevalence) or epidemiology) or 筛查)	44332
#1	((((脊柱侧凸) or 脊柱侧弯) or 脊柱畸形) or scoliosis)	2720

Table S2 STROBE statement for the quality of observational studies included

	Item No	Recommendation
**Title and abstract**	1	(a) Indicate the study's design with a commonly used term in the title or the abstract
		(b) Provide in the abstract an informative and balanced summary of what was done and what was found
**Introduction**		
Background/rationale	2	Explain the scientific background and rationale for the investigation being reported
Objectives	3	State specific objectives, including any prespecified hypotheses
**Methods**		
Study design	4	Present key elements of study design early in the paper
Setting	5	Describe the setting, locations, and relevant dates, including periods of recruitment, exposure, follow-up, and data collection
Participants	6	(a) Give the eligibility criteria, and the sources and methods of selection of participants
Variables	7	Clearly define all outcomes, exposures, predictors, potential confounders, and effect modifiers. Give diagnostic criteria, if applicable
Data sources/ measurement	8*	For each variable of interest, give sources of data and details of methods of assessment (measurement). Describe comparability of assessment methods if there is more than one group
Bias	9	Describe any efforts to address potential sources of bias
Study size	10	Explain how the study size was arrived at
Quantitative variables	11	Explain how quantitative variables were handled in the analyses. If applicable, describe which groupings were chosen and why
Statistical methods	12	(a) Describe all statistical methods, including those used to control for confounding
		(b) Describe any methods used to examine subgroups and interactions
		(c) Explain how missing data were addressed
		(d) If applicable, describe analytical methods taking account of

		sampling strategy
		(e) Describe any sensitivity analyses
**Results**		
Participants	13*	(a) Report numbers of individuals at each stage of study—eg numbers potentially eligible, examined for eligibility, confirmed eligible, included in the study, completing follow-up, and analysed
		(b) Give reasons for non-participation at each stage
		(c) Consider use of a flow diagram
Descriptive data	14*	(a) Give characteristics of study participants (eg demographic, clinical, social) and information on exposures and potential confounders
		(b) Indicate number of participants with missing data for each variable of interest
Outcome data	15*	Report numbers of outcome events or summary measures
Main results	16	(a) Give unadjusted estimates and, if applicable, confounder-adjusted estimates and their precision (eg, 95% confidence interval). Make clear which confounders were adjusted for and why they were included
		(b) Report category boundaries when continuous variables were categorized
		(c) If relevant, consider translating estimates of relative risk into absolute risk for a meaningful time period
Other analyses	17	Report other analyses done—eg analyses of subgroups and interactions, and sensitivity analyses
**Discussion**		
Key results	18	Summarise key results with reference to study objectives
Limitations	19	Discuss limitations of the study, taking into account sources of potential bias or imprecision. Discuss both direction and magnitude of any potential bias
Interpretation	20	Give a cautious overall interpretation of results considering objectives, limitations, multiplicity of analyses, results from similar studies, and other relevant evidence
Generalizability	21	Discuss the generalizability (external validity) of the study results

Other information		
Funding	22	Give the source of funding and the role of the funders for the present study and, if applicable, for the original study on which the present article is based

Footnote: *Give information separately for exposed and unexposed groups.

An Explanation and Elaboration article discusses each checklist item and gives methodological background and published examples of transparent reporting. The STROBE checklist is best used in conjunction with this article (freely available on the Web sites of PLoS Medicine at http://www.plosmedicine.org/, Annals of Internal Medicine at http://www.annals.org/, and Epidemiology at http://www.epidem.com/). Information on the STROBE Initiative is available at www.strobe-statement.org.

Table S3 PRISMA checklist for meta-analysis

Section/topic	#	Checklist item	Reported on page #
**TITLE**			1
Title	1	Identify the report as a systematic review, meta-analysis, or both.	1
**ABSTRACT**			2
Structured summary	2	Provide a structured summary including, as applicable: background; objectives; data sources; study eligibility criteria, participants, and interventions; study appraisal and synthesis methods; results; limitations; conclusions and implications of key findings; systematic review registration number.	2
**INTRODUCTION**			3
Rationale	3	Describe the rationale for the review in the context of what is already known.	3
Objectives	4	Provide an explicit statement of questions being addressed with reference to participants, interventions, comparisons, outcomes, and study design (PICOS).	3
**METHODS**			3-5
Protocol and registration	5	Indicate if a review protocol exists, if and where it can be accessed (e.g., Web address), and, if available, provide registration information including registration number.	3
Eligibility criteria	6	Specify study characteristics (e.g., PICOS, length of follow-up) and report characteristics (e.g., years considered, language, publication status) used as criteria for eligibility, giving rationale.	4
Information sources	7	Describe all information sources (e.g., databases with dates of coverage, contact with study authors to identify additional studies) in the search and date last searched.	4
Search	8	Present full electronic search strategy for at least one database, including any limits used, such that it could be repeated.	4
Study selection	9	State the process for selecting studies (i.e., screening, eligibility, included in systematic review, and, if applicable, included in the meta-analysis).	4
Data collection process	10	Describe method of data extraction from reports (e.g., piloted forms, independently, in duplicate) and any processes for obtaining and confirming data from investigators.	4
Data items	11	List and define all variables for which data were sought (e.g., PICOS, funding sources) and any assumptions and simplifications made.	4
Risk of bias in individual studies	12	Describe methods used for assessing risk of bias of individual studies (including specification of whether this was done at the study or outcome level), and how this information is to be used in any data synthesis.	4
Summary measures	13	State the principal summary measures (e.g., risk ratio, difference in means).	5
Synthesis of results	14	Describe the methods of handling data and combining results of studies, if done, including measures of consistency (e.g., I^2) for each meta-analysis.	5

Section/topic	#	Checklist item	Reported on page #
Risk of bias across studies	15	Specify any assessment of risk of bias that may affect the cumulative evidence (e.g., publication bias, selective reporting within studies).	5
Additional analyses	16	Describe methods of additional analyses (e.g., sensitivity or subgroup analyses, meta-regression), if done, indicating which were pre-specified.	5
**RESULTS**			5-7
Study selection	17	Give numbers of studies screened, assessed for eligibility, and included in the review, with reasons for exclusions at each stage, ideally with a flow diagram.	5
Study characteristics	18	For each study, present characteristics for which data were extracted (e.g., study size, PICOS, follow-up period) and provide the citations.	6
Risk of bias within studies	19	Present data on risk of bias of each study and, if available, any outcome level assessment (see item 12).	6
Results of individual studies	20	For all outcomes considered (benefits or harms), present, for each study: (a) simple summary data for each intervention group (b) effect estimates and confidence intervals, ideally with a forest plot.	6
Synthesis of results	21	Present results of each meta-analysis done, including confidence intervals and measures of consistency.	6
Risk of bias across studies	22	Present results of any assessment of risk of bias across studies (see Item 15).	6
Additional analysis	23	Give results of additional analyses, if done (e.g., sensitivity or subgroup analyses, meta-regression [see Item 16]).	7
**DISCUSSION**			7-10
Summary of evidence	24	Summarize the main findings including the strength of evidence for each main outcome; consider their relevance to key groups (e.g., healthcare providers, users, and policy makers).	7
Limitations	25	Discuss limitations at study and outcome level (e.g., risk of bias), and at review-level (e.g., incomplete retrieval of identified research, reporting bias).	9
Conclusions	26	Provide a general interpretation of the results in the context of other evidence, and implications for future research.	10
**FUNDING**			10
Funding	27	Describe sources of funding for the systematic review and other support (e.g., supply of data); role of funders for the systematic review.	10

From: Moher D, Liberati A, Tetzlaff J, Altman DG, The PRISMA Group (2009). Preferred Reporting Items for Systematic Reviews and Meta-Analyses: The PRISMA Statement. PLoS Med 6(7): e1000097. doi:10.1371/journal.pmed1000097

For more information, visit: **www.prisma-statement.org**.

Table S4 The types of measurements for scoliosis screening

No	Primary screening		Secondary screening		Third Screening	
	**Method**	**N (%)**	**Method**	**N (%)**	**Method**	**N (%)**
1	PE+FBT	48 (62.3)	Moire photograph	17 (22.1)	X-ray	62 (80.5)
2	PE+ATR	23 (29.9)	FBT+ATR (>5°)	13(16.9)	NA	15 (19.5)
3	PE+Plumb	1 (1.30)	Movement+Prone test	7 (9.09)		
4	Moire photograph	2 (2.60)	FBT+ATR (>4°)	4 (5.20)		
5	Questionaire	1 (1.30)	Specific inquiry	2 (2.60)		
6	NA	2 (2.60)	FBT+ATR (>7°)	1 (1.30)		
7			FBT+ATR (>3°) by Mesh Lab (3D)	1 (1.30)		
8			PE	1 (1.30)		
9			Ponint line	1 (1.30)		
10			NA	30 (40.0)		

Footnote: PE: physical examination; FBT: forward bend test; ATR: angle of trunk rotation

[a]: PE+ATR (>5°): means the participant was positively diagnosed when the scoliometer was with a value larger than 5°, the similar definition to PE+ATR (>4°) and PE+ATR (>7°)

Table S5 Quality assessments of the included studies according to the STROBE statement

No	Author & Year	1a[a]	1b	2	3	4	5	6	7	8	9	10	11	12a	12b	12c	12d	12e	13a	13b	13c	14a	14b	15	16a	16b	16c	17	18	19	20	21	22
1	Qiu, 2022	Y	Y	Y	Y	Y	Y	Y	Y	Y	N	N	Y	Y	U	N	N	N	Y	N	N	Y	N	Y	Y	N	U	Y	Y	N	Y	Y	N
2	Chen et al, 2021	Y	Y	Y	Y	Y	Y	Y	Y	U	N	N	Y	Y	N	N	N	N	Y	N	N	Y	N	Y	U	N	Y	Y	Y	N	Y	Y	Y
3	Sun et al, 2021	Y	Y	Y	Y	Y	Y	Y	Y	Y	U	N	Y	Y	N	N	N	N	Y	N	N	Y	N	N	Y	Y	Y	Y	Y	N	Y	Y	Y
4	Cai et al, 2021	Y	Y	Y	Y	Y	Y	Y	Y	Y	N	Y	Y	Y	N	N	U	Y	Y	U	N	Y	Y	Y	Y	Y	Y	Y	Y	Y	Y	Y	Y
5	Li et al, 2021	Y	Y	Y	Y	Y	Y	Y	Y	N	N	N	U	Y	N	Y	N	N	Y	Y	N	Y	Y	U	Y	N	N	N	Y	N	Y	Y	N
6	Wen et al, 2021	Y	Y	Y	Y	Y	Y	Y	Y	Y	U	N	U	U	N	N	N	N	Y	N	N	Y	N	N	Y	N	U	Y	Y	N	Y	Y	N
7	Ding et al, 2020	Y	Y	Y	Y	Y	Y	Y	Y	Y	N	Y	Y	Y	N	N	N	N	Y	N	N	Y	N	Y	Y	Y	Y	Y	Y	Y	Y	Y	Y
8	Yang et al, 2020	Y	Y	Y	Y	Y	Y	Y	Y	Y	Y	Y	Y	Y	Y	U	U	U	Y	N	N	Y	N	Y	Y	Y	Y	Y	Y	Y	Y	Y	U
9	Xia et al, 2019	Y	Y	Y	Y	Y	Y	Y	Y	Y	N	U	Y	Y	N	N	U	N	Y	U	N	U	N	Y	U	U	N	Y	N	Y	N	U	Y
10	Zeng, 2019	Y	Y	Y	Y	Y	Y	Y	Y	Y	N	N	Y	Y	Y	N	N	N	Y	N	N	Y	N	Y	Y	Y	Y	Y	Y	Y	N	N	N
11	Wang et al, 2018	Y	N	Y	Y	Y	Y	Y	Y	Y	N	N	Y	Y	N	N	N	N	Y	Y	N	Y	Y	Y	U	Y	N	Y	Y	Y	Y	Y	Y
12	Wei et al, 2018	Y	Y	Y	Y	Y	Y	Y	Y	Y	N	N	Y	Y	N	N	N	N	Y	N	N	Y	Y	Y	Y	Y	N	Y	Y	Y	Y	Y	Y
13	Li et al, 2018	Y	Y	Y	Y	Y	Y	Y	Y	Y	N	N	U	Y	U	N	N	N	Y	N	N	U	N	Y	N	Y	N	U	Y	U	Y	Y	Y
14	Du et al, 2018	Y	Y	Y	Y	Y	Y	Y	Y	Y	Y	Y	Y	Y	Y	N	N	N	Y	N	N	Y	N	Y	Y	Y	Y	Y	Y	Y	N	N	Y
15	Li et al, 2018	Y	Y	Y	Y	Y	Y	Y	Y	Y	N	N	Y	Y	N	N	N	N	Y	N	N	Y	N	Y	Y	Y	N	Y	Y	N	N	N	N
16	Deng et al, 2018	Y	Y	Y	Y	Y	Y	Y	Y	Y	N	Y	Y	N	Y	N	Y	N	Y	N	N	Y	N	Y	N	Y	Y	N	Y	Y	Y	Y	N
17	Wang et al, 2018	Y	Y	Y	Y	Y	Y	Y	Y	Y	N	Y	Y	N	N	N	Y	N	Y	N	N	Y	N	N	Y	N	N	N	Y	N	N	Y	Y
18	He et al, 2018	N	N	Y	N	Y	N	N	N	Y	N	N	Y	N	N	N	N	N	Y	N	N	Y	N	N	N	N	N	N	Y	N	Y	Y	Y
19	Tang et al, 2017	Y	Y	U	Y	Y	Y	Y	Y	U	N	N	U	Y	U	N	U	N	Y	N	N	Y	N	Y	N	U	N	N	Y	Y	Y	U	N
20	Miao et al, 2017	Y	Y	Y	Y	Y	Y	Y	Y	U	N	U	U	Y	N	N	U	N	Y	U	N	Y	N	Y	U	Y	N	U	Y	U	Y	Y	Y
21	Nie et al, 2017	Y	Y	Y	Y	Y	Y	Y	Y	Y	U	Y	Y	Y	Y	N	Y	N	U	U	N	U	N	Y	N	Y	N	Y	Y	Y	Y	Y	Y
22	Li et al, 2017	Y	Y	Y	Y	Y	Y	Y	Y	Y	Y	Y	Y	Y	N	N	N	N	Y	U	N	Y	N	Y	Y	N	N	U	Y	Y	Y	Y	Y

23	Deng et al, 2017	Y	Y	Y	Y	Y	Y	Y	Y	Y	N	N	N	Y	N	N	N	N	Y	N	N	Y	Y	Y	Y	Y	Y	N	Y	Y	Y	Y	Y
24	Hu et al, 2017	Y	Y	Y	Y	Y	Y	Y	Y	Y	Y	Y	Y	Y	Y	N	N	N	Y	N	N	Y	N	Y	Y	Y	Y	Y	Y	Y	N	N	Y
25	Han et al, 2017	Y	N	Y	Y	N	N	N	N	Y	N	N	N	N	N	N	N	N	N	N	N	Y	N	N	N	N	N	N	Y	N	Y	Y	Y
26	Du et al, 2016	Y	Y	Y	Y	Y	Y	Y	Y	Y	U	U	Y	Y	Y	U	U	Y	Y	Y	N	Y	Y	Y	U	Y	N	Y	Y	Y	Y	Y	Y
27	Zheng et al, 2016	Y	Y	Y	Y	Y	Y	Y	Y	Y	Y	Y	Y	Y	Y	U	Y	U	Y	Y	N	Y	U	Y	Y	Y	Y	Y	Y	Y	Y	Y	Y
28	He et al, 2016	Y	Y	Y	Y	Y	Y	Y	Y	Y	N	N	Y	N	N	N	U	N	Y	U	N	U	U	Y	N	Y	U	U	Y	N	Y	U	N
29	Fan et al, 2016	Y	Y	Y	Y	Y	Y	Y	Y	Y	Y	Y	Y	Y	Y	U	U	U	Y	N	N	Y	N	Y	Y	Y	Y	Y	Y	Y	Y	Y	Y
30	Huang et al, 2016	Y	Y	Y	Y	Y	Y	Y	Y	Y	N	N	Y	Y	N	N	Y	N	Y	N	N	Y	N	Y	N	Y	N	N	Y	N	Y	Y	N
31	Chen et al, 2016	Y	Y	Y	Y	Y	Y	N	Y	Y	N	N	Y	N	N	N	N	N	N	N	N	Y	N	N	N	N	N	N	Y	N	Y	Y	Y
32	Ke et al, 2015	Y	Y	Y	Y	Y	Y	Y	Y	Y	N	N	U	Y	U	N	U	N	Y	N	N	Y	N	Y	U	Y	N	N	Y	U	Y	N	N
33	Ma et al, 2015	Y	Y	Y	Y	Y	Y	Y	Y	Y	N	N	N	N	N	N	Y	N	Y	N	N	Y	N	Y	N	N	N	N	Y	Y	Y	Y	Y
34	Chen et al, 2015	Y	Y	Y	Y	Y	Y	N	Y	Y	N	N	Y	N	N	N	N	N	N	N	N	Y	N	N	N	N	N	N	Y	N	Y	Y	Y
35	Yu et al, 2014	Y	Y	Y	Y	Y	Y	Y	Y	Y	N	N	Y	Y	N	N	N	N	Y	N	N	Y	N	Y	Y	Y	Y	N	Y	Y	N	N	Y
36	Zhao et al, 2014	Y	Y	Y	Y	Y	Y	Y	Y	Y	N	N	Y	Y	N	N	N	N	Y	U	U	Y	N	Y	Y	Y	Y	N	Y	N	N	N	Y
37	Ren et al, 2014	Y	Y	Y	N	Y	Y	N	Y	N	N	N	Y	N	N	N	N	N	Y	N	N	Y	N	N	N	Y	N	N	Y	N	Y	Y	N
38	Wang et al, 2013	Y	Y	Y	Y	Y	Y	Y	Y	Y	N	Y	U	Y	Y	N	U	N	Y	U	N	Y	N	Y	N	Y	N	U	Y	Y	Y	U	Y
39	Ke et al, 2012	Y	Y	Y	Y	Y	Y	Y	Y	Y	N	N	Y	Y	N	N	N	N	Y	N	N	Y	N	Y	Y	Y	Y	Y	Y	Y	N	N	Y
40	Chen et al, 2012	Y	Y	Y	Y	Y	Y	Y	Y	Y	N	N	Y	Y	Y	U	N	N	Y	N	N	Y	N	Y	Y	Y	Y	Y	Y	Y	Y	N	Y
41	Zhang et al, 2011	Y	Y	Y	Y	Y	Y	Y	Y	Y	N	Y	U	Y	U	N	Y	N	Y	N	N	Y	U	Y	N	Y	N	U	Y	N	U	Y	Y
42	Liu et al, 2011	Y	Y	Y	Y	Y	Y	Y	Y	Y	N	N	U	U	N	N	U	N	Y	Y	N	Y	U	Y	U	Y	U	Y	Y	U	Y	Y	Y
43	Huang et al, 2011	Y	Y	Y	Y	Y	Y	Y	Y	Y	N	N	Y	Y	N	N	N	N	Y	N	N	Y	N	Y	Y	Y	Y	N	Y	Y	N	N	Y
44	Tang et al, 2011	Y	Y	Y	Y	Y	Y	Y	Y	Y	N	N	Y	Y	Y	N	N	N	Y	N	N	Y	N	Y	Y	Y	Y	Y	Y	Y	N	N	Y
45	Li et al, 2011	Y	Y	Y	Y	Y	Y	Y	Y	Y	N	N	Y	Y	Y	Y	N	N	Y	N	N	Y	N	Y	Y	Y	Y	Y	Y	Y	Y	N	N
46	Chen et al, 2010	Y	Y	Y	Y	Y	Y	Y	Y	Y	N	U	N	Y	Y	N	N	N	Y	N	N	U	N	Y	N	Y	N	Y	Y	N	Y	Y	N

47	Lu et al, 2010	Y	Y	Y	Y	Y	Y	Y	U	Y	N	U	U	Y	N	N	U	N	Y	U	N	Y	N	Y	N	Y	N	N	Y	N	Y	Y	Y
48	Yu et al, 2010	Y	Y	Y	Y	Y	Y	Y	Y	Y	U	Y	Y	U	N	N	N	N	Y	N	N	Y	N	Y	Y	N	N	N	Y	N	Y	Y	N
49	Du et al, 2010	Y	Y	Y	Y	Y	Y	Y	Y	Y	Y	N	Y	Y	Y	N	N	N	Y	N	N	Y	N	Y	Y	Y	Y	Y	Y	Y	N	N	Y
50	Dong et al, 2009	Y	Y	Y	Y	Y	Y	Y	Y	Y	U	Y	Y	Y	Y	N	N	N	Y	N	N	Y	N	Y	Y	Y	Y	U	Y	N	Y	N	N
51	Zhou et al, 2008	Y	Y	Y	Y	Y	Y	Y	Y	Y	N	Y	Y	Y	N	N	N	N	Y	N	N	Y	N	Y	Y	Y	Y	N	Y	N	Y	Y	N
52	Zhang et al, 2008	Y	Y	Y	Y	Y	Y	Y	Y	Y	N	Y	Y	Y	N	N	N	N	Y	N	N	Y	N	Y	Y	Y	Y	Y	Y	Y	Y	Y	N
53	Sun et al, 2008	N	N	N	N	Y	Y	N	Y	Y	N	N	N	Y	N	N	N	N	Y	N	N	Y	N	N	N	Y	N	N	Y	N	N	Y	Y
54	Wang et al, 2007	Y	Y	Y	Y	Y	Y	Y	U	U	N	N	N	U	N	N	Y	N	Y	Y	N	Y	U	Y	U	Y	N	Y	Y	U	Y	Y	Y
55	Yu et al, 2006	U	N	Y	Y	Y	Y	Y	Y	Y	N	N	U	Y	N	N	U	N	Y	N	N	Y	N	Y	N	Y	N	Y	Y	U	U	Y	Y
56	Cheng et al, 2006	Y	Y	Y	N	Y	Y	N	N	Y	N	Y	Y	N	N	N	Y	N	Y	N	N	Y	N	Y	N	N	N	N	Y	N	N	Y	N
57	Liang et al, 2005	Y	Y	Y	Y	Y	Y	Y	Y	Y	N	N	Y	Y	N	N	N	N	Y	N	N	Y	N	Y	Y	Y	Y	Y	Y	Y	N	N	N
58	Gao et al, 2004	Y	Y	Y	Y	Y	Y	Y	Y	Y	N	N	Y	N	N	N	U	N	Y	N	N	Y	U	Y	N	Y	N	U	N	U	Y	Y	N
59	Meng et al, 2003	Y	Y	Y	Y	Y	Y	Y	U	Y	U	N	N	Y	U	N	N	N	Y	N	N	U	N	N	N	Y	N	Y	Y	U	U	Y	N
60	Zhang et al, 2003	N	N	Y	Y	Y	Y	Y	Y	Y	N	N	N	N	N	N	N	N	Y	N	N	N	N	Y	N	N	N	N	Y	Y	Y	Y	N
61	Liu et al, 2002	Y	Y	Y	Y	Y	Y	Y	Y	Y	N	N	Y	Y	N	N	N	N	Y	N	N	Y	N	Y	Y	Y	U	U	Y	Y	U	N	Y
62	Liang et al, 2002	Y	Y	Y	N	N	Y	Y	N	N	N	N	N	N	N	N	N	N	Y	N	N	Y	N	Y	N	Y	N	N	Y	N	Y	Y	N
63	Li et al, 2001	Y	Y	Y	Y	Y	Y	Y	Y	Y	U	U	Y	Y	N	N	N	N	Y	N	N	Y	U	Y	Y	Y	Y	U	Y	Y	Y	N	Y
64	Li et al, 1999	Y	Y	Y	Y	Y	Y	Y	Y	Y	N	N	Y	Y	N	N	N	N	Y	N	N	Y	N	Y	Y	Y	Y	N	Y	Y	N	N	N
65	Li et al, 1999	Y	Y	Y	Y	Y	Y	Y	Y	Y	Y	Y	Y	Y	Y	U	U	U	Y	N	N	Y	N	Y	Y	Y	Y	Y	Y	Y	Y	Y	Y
66	Wang et al, 1998	Y	Y	Y	Y	Y	Y	Y	Y	Y	N	N	Y	Y	N	N	N	N	Y	N	N	Y	N	Y	Y	N	N	Y	Y	Y	N	N	N
67	Wang et al, 1996	Y	Y	Y	Y	Y	Y	Y	U	Y	N	N	U	U	N	N	U	N	Y	Y	N	Y	N	Y	U	U	N	Y	Y	N	Y	Y	N
68	Zhao et al, 1996	Y	N	Y	Y	Y	Y	Y	U	U	U	N	U	U	N	N	N	N	Y	U	N	Y	N	Y	N	Y	N	Y	Y	N	U	U	N
69	Yu et al, 1995	Y	Y	Y	Y	Y	Y	Y	Y	Y	N	N	U	N	U	N	N	N	Y	U	N	Y	N	Y	U	U	N	Y	Y	N	Y	Y	N
70	Ma et al, 1995	Y	Y	Y	Y	Y	Y	Y	Y	U	N	N	U	N	N	N	N	N	Y	N	N	U	N	U	N	U	N	U	Y	N	U	Y	N

71	Jiang et al, 1994	Y	Y	Y	Y	Y	Y	Y	Y	Y	N	Y	U	N	N	N	Y	N	Y	N	N	Y	N	Y	N	U	N	N	Y	N	Y	Y	N
72	Chen et al, 1990	Y	U	U	Y	Y	Y	N	U	Y	N	N	Y	N	N	U	U	N	Y	Y	N	Y	Y	Y	Y	Y	N	Y	Y	Y	Y	Y	N
73	Cao et al, 1989	Y	N	U	Y	Y	Y	U	U	N	N	N	N	N	N	N	N	N	Y	N	N	N	N	Y	N	N	N	Y	Y	N	N	U	N
74	Zhang et al, 1988	Y	Y	Y	Y	Y	Y	Y	U	Y	N	N	Y	U	N	N	N	N	Y	Y	Y	Y	N	Y	U	U	N	Y	Y	N	Y	U	N
75	Tan et al, 1987	Y	U	Y	Y	Y	U	U	Y	U	N	N	Y	U	N	N	U	N	Y	N	N	U	N	Y	U	N	N	Y	Y	U	Y	U	N
76	Wang et al, 1985	Y	Y	Y	Y	Y	Y	Y	U	Y	N	N	U	N	N	N	N	N	Y	U	N	Y	N	Y	U	Y	U	Y	Y	U	N	U	N
77	Pin et al, 1985	Y	Y	Y	Y	Y	Y	Y	Y	Y	U	N	Y	Y	N	N	N	N	Y	U	N	Y	Y	Y	Y	Y	U	U	Y	Y	Y	Y	N

Footnote: [a]The mean of each number is explained in Table S2

[b]Y: yes; N: no; U: unclear

Table S6 Number of scoliosis cases in different age groups

Author & Year	Scoliosis cases in different age groups (No.)												Participants in different age groups (No.)												
	7~	8~	9~	10~	11~	12~	13~	14~	15~	16~	17~	18	6~	7~	8~	9~	10~	11~	12~	13~	14~	15~	16~	17~	18
Ding et al., 2020						12	59	64	40	32									3432	3317	4256	3845	3668		
Xia et al., 2019	0	0	4	3	1	9	6	6	4					393	392	423	416	399	547	520	399	424			
Zeng, 2019	6	12	12	54	24	66	96	90	84	78	12			1542	2850	5418	10572	11370	14184	18456	15168	13830	7584	3114	
Wang et al., 2018							23	9	12	12	2	3								5991	5587	4572	2759	3004	3184
Li et al., 2018					12	13	20	23	27	31								2653	2464	2566	2581	2413	2570		
Li et al., 2018						6	480	274	300	71	77	30							455	12027	11701	10922	2014	2236	1903
Deng et al., 2018						23	23	25	25	27									978	1003	1060	1008	1077		
Miao et al., 2017				7	16	40	35	49	46	5	2						8694	14927	12960	13137	11763	5173	509	159	
Du et al., 2016	16	12	24	23	16	17	19	18	8	10	5		163	873	853	903	849	746	592	601	529	250	247	218	
Chen et al., 2016	0	0	8	18	14	26	78	68	61	32	33	22		908	1385	1517	1751	1821	2297	4993	4227	3684	1942	1896	1469
Ke et al., 2015						6	31	15	43	50	15	1							2708	3293	800	3768	4107	786	205
Yu et al., 2014	3	6	7	10	12	22	55	54	46	11	10	4		1167	1900	1932	2153	2179	3006	5792	5000	4153	1057	890	303
Zhao et al., 2014	2	2	3	4	8	9	18	20	19					321	424	532	611	772	913	1620	1752	1406			
Ren et al., 2014	1	2	2	9	4	11	16	15	14	13	2			257	475	903	1762	1895	2364	3076	2528	2305	1264	519	
Ke et al., 2012	2	3	5	5	9	19	47	43	23					910	1211	1157	1174	1305	2153	5158	4109	1621			
Chen et al., 2012	5	8	7	9	11	15	21	24	20	16				1892	1966	2123	1846	1965	1934	1923	2102	1834	2061		
Zhang et al., 2011	0	0	1	2	0	5	2							120	120	120	120	120	120	120					
Liu et al., 2011	34	38	33	34	36	38	48	54	57	32			1559	2347	2489	2170	2225	2406	2472	2478	2372	2427	1417		
Huang et al., 2011	0	3	1	2	1	11	21	31	40	35	35	19		1181	1277	1295	1259	1234	1298	3888	3886	4252	3711	3497	2114
Chen et al., 2010	0	0	1	1	2	4	6	11	14	2				1124	1242	1221	1322	1297	1727	1457	1491	1175	201		
Lu et al., 2010	19	45	46	26	32	26	50	51	40					1556	2767	2429	1695	2231	1408	2131	1933	1374			

Dong et al., 2009			3	2	7	8	16	17	11							976	864	1029	953	2037	2386	1874			
Zhou et al., 2008	0	0	1	3	3	8	49	37	37	33	35	27		352	954	1264	1450	1564	1763	5520	4382	4272	3623	3828	3032
Zhang et al., 2008	1	1	1	2	3	6	12	20	32	32	33	15		393	490	497	529	562	1079	2329	3409	3628	3709	3000	1487
Wang et al., 2007	0	0	1	1	1	3	4	9	11	7	21	6	2610	3094	3680	3538	3738	3672	3133	4672	5740	6090	8143	7719	1559
Cheng et al., 2006	20	46	48	26	32	26	51	53	41					2283	4061	3567	2489	3275	2068	3129	2836	2017			
Gao et al., 2004	2	4	4	6	8	9	10	12	10					995	965	969	953	956	949	960	950	955			
Liu et al., 2002	11	15	23	34	40	73	147	144	117	24	16	9		4742	6106	6086	7062	6993	9575	16356	14528	11477	2302	1461	858
Li et al., 2001	5	6	11	12	20	44	108	100	55					1637	2177	2080	2110	2347	3871	9274	7388	2914			
Li et al., 1999	4	7	7	9	11	14	19	21	19					2023	2154	2218	1907	1914	2017	2119	1943	2274			
Zhao et al., 1996	42	43	30	60	78	50	43	72	84				960	980	1020	1210	860	970	950	1070	1010	1043			
Ma et al., 1995	10	12	15	20	27	31	35	36	34	31				1346	1451	1604	1657	1758	1779	1891	1876	1741	1555		
Jiang et al., 1994	114	90	68	48	48	43							1283	8504	7021	5591	4936	5577	4091						
Zhang et al., 1988	32	32	39	41	61	153	235	301	313					1160	978	889	989	1187	2682	3924	4452	4155			
Pin et al., 1985	25	27	22	15	21	11	18	12	2				571	975	1193	1048	1065	937	903	1041	343	89			

Table S7 Details on the cause, distribution, and treatment for scoliosis

Author & Year	Other types of scoliosis			Cobb angle			Latitude	Altitude	Remarks
	CS	NMS	Others	10-19°	20-39°	≥40°	(°)	(m)	
Ding et al., 2020	25	2	0	184	33	17	34.75	234.28	The prevalence in rural areas was higher than in urban areas
Yang et al., 2020							22.54	87.05	Children and adolescents had a high prevalence of incorrect posture, with girls and older students being an especially high-risk group
Xia et al., 2019	1	0	0	27	6	0	34.23	2.19	
Zeng, 2019	30	18	6 (traumatic)	414	78	42	23.07	170.97	-
Wang et al., 2018				43	17	1	39.9	368.59	
Wei et al., 2018							30.7	662.18	Peaked at 13–15 years; prevalence was higher in those with lower BMI and bi- or dextro-manual preference
Li et al., 2018				92	23	11	22.54	87.05	-
Du et al., 2018	0	0	0	115	19	1	23.37	55.44	-
Li et al., 2018				1140	90	8	22.52	22.78	-
Deng et al., 2018	11	0	5	110	11	2	30.05	4188.7	The prevalence in family history of spinal disease, agricultural population, and poor sitting posture was higher; family history of spinal disease was an independent risk factor
Wang et al., 2018				15	5	3	25.04	2100.17	Back PEI questionnaire was used in the first screening. The risk factors of IS were competitive sports, sleep time over 10 h, and sleeping position. The validity of ATR>5° was 91.3% and that of ATR>5° was 78.3%
He et al., 2018	13	2	0				36.62	3106.83	The prevalence of IS higher than reported; no difference in sex for IS; Han in higher prevalence, but with no difference to Hui and Tibetan
Tang et al., 2017							32.23	2.19	94 and 8 cases had Cobb angle between 10 and 25° and between 25 and 45°, while in 8 cases, it was difficult to classify the Cobb angle.
Miao et al., 2017	4	1	0	172	23	5	31.57	17.18	There was no significance on scoliosis prevalence between rural and urban areas
Nie et al., 2017							28.45	627.38	Network screening can improve the detection rate of AIS, and can clarify the pathological type and possible progress of scoliosis cases
Li et al., 2017				16	8	5	33.57	65.29	The awareness rate and treatment rate of congenital scoliosis were low
Deng et al., 2017							32.01	348.39	Regular activity and exercise were protective factors against scoliosis
Hu et al., 2017	6	2	0	113	26	4	22.54	87.05	Regular outpatient clinic for Cobb angle 10–19° and brace for Cobb angle >20° and no surgery
Han et al., 2017							36.05	2087.94	-
Du et al., 2016				162	10	0	31.23	2.19	Epidemiology of scoliosis had regional variation, and genetic differences might contribute to such differences
Zheng et al., 2016							31.57	17.18	Female sex, use of single-shoulder bags, and long duration of computer use were risk factors

He et al., 2016				120	34	0	24.9	451.19	-
Fan et al., 2016				3831	1261	43	23.12	106.33	Low BMI may be a risk factor for IS
Huang et al., 2016							25.04	2100.17	The prevalence increased by age
Chen et al., 2016	6	3	0	299	54	7	34.26	1023.65	-
Ke et al., 2015	3	2	0	97	58	6	32.2	23.04	Female patients were more likely to progress to severe deformity than male patients
Ma et al., 2015							18.24	187.26	
Chen et al., 2015				329	56	8	34.26	1023.65	-
Fong et al., 2015							22.26	25.57	In the 5-year follow-up from 1996 to 2000, the AIS (Cobb>20) increased by 0.23%
Yu et al., 2014	7	3	0	187	53	0	23.12	106.33	-
Zhao et al., 2014	3	1	0	72	11	2	23.12	106.33	-
Ren et al., 2014	5	3	1(traumatic)				29.35	370.83	69 cases with Cobb angle between 10 and 20°, 13 cases with Cobb angle between 21 and 30°, and 7 cases with Cobb angle between 31 and 50°
Wang et al., 2013							28	358.52	The prevalence in rural areas was higher than in urban areas
Ke et al., 2012	3	3	0	129	21	6	23.02	25.27	The prevalence increased by age; female patients were more likely to progress to severe deformity than male patients
Chen et al., 2012				103	28	5	21.85	144.45	-
Zhang et al., 2011							40.82	695.14	Mongolian, Han, and Hui pupils had no significant difference in terms of scoliosis. Bad habits in leaning and living have considerable influence on scoliosis
Liu et al., 2011	30	13	12				45.75	251.23	Thermosetting plastic brace is an effective treatment for AIS
Huang et al., 2011	19	0	0	105	92	14	23.12	106.33	-
Tang et al., 2011				113	26	4	22.54	87.05	Adam’s bending test and scoliometry were simple and effective
Li et al., 2011	4	0	1				22.52	22.78	-
Chen et al., 2010	2	1	0	26	9	3	41.11	1381.08	There was large heterogeneity world-widely
Lu et al., 2010	21	0	3	273	44	8	44.75	250.23	Prevalence of scoliosis increased with age between 7 and 15 years and peaked between 13 and 15 years
Yu et al., 2010							24.5	162.8	Low BMI, wrong posture, uncomfortable mattress, and heavy schoolbag were risk factors for scoliosis
Du et al., 2010	3	1	0	76	17	1	23.02	25.27	Regular outpatient clinic for Cobb angle 10–19° and brace for Cobb angle >20° and no surgery
Dong et al., 2009	2	0	0	49	10	5	28.67	38.9	First screening was by physical examination and a self-made scoliometer

Zhou et al., 2008	4	1	0	186	47	1	24.9	451.19	-
Zhang et al., 2008	4	1	0	129	21	8	24.9	451.19	The prevalence increased by age; female patients were more likely to progress to severe deformity than male patients
Sun et al., 2008	8	0	1	160	20	4	26.58	1715.44	-
Wu et al., 2008							24	10	Girls in grades 4-6 were higher than grades 1-3, lower in rural
Wang et al., 2007	4	1	1	31	20	8	39.9	368.59	
Yu et al., 2006							30.27	274.17	Myopia, lack of calcium supplement, single-shoulder bag use, and less activity might be related to higher prevalence of scoliosis
Cheng et al., 2006	21	1	0	273	59	11	34.26	1023.65	Regular exercise for 15 cases with CS between 10 and 25°, neo-plastic brace for 5 cases with CS between 25 and 40°, and 1 case with CS >40° needed surgery
Liang et al., 2005	5	0	0	57	13	2	23.05	213.06	Regular exercise for Cobb angle 10–19° and brace for Cobb angle 20–39° and no surgery
Gao et al., 2004	2	0	0	58	6	1	32.01	4.01	"Point Line" was a simple, quick, and effective way for scoliosis investigation
Meng et al., 2003	17	0	9	224	27	0	39.5	14.2	-
Zhang et al., 2003							20.03	41.85	Serum melatonin levels may contribute to the pathogenesis of IS
Liu et al., 2002				569	62	22	23.12	106.33	-
Liang et al., 2002							29.66	4828.08	No difference in sex; in addition to congenital diseases, chest diseases in young students were mainly affected by scoliosis and infectious diseases (mostly in tuberculous infection)
Li et al., 2001	6	5	0	321	32	8	23.12	106.33	-
Lee et al., 2001							22.68	13	The positive predictive value was 17.3%
Wang et al., 2000							23.17	25	The prevalence was related to height and weight, prone to happening in elongated figure
Li et al., 1999				68	4	2	24.8	414.77	Regular activity and exercise for Cobb angle 10–19°; brace, for 20–45°; and surgery, for >45°
Li et al., 1999	5	0	0	93	14	5	22.52	22.78	Regular outpatient clinic for Cobb angle 10–19°, exercise and traction for Cobb angle 20–39°, and surgery for Cobb angle >40°
Lee et al., 1999							25.03	9	Height was a risk factor for the prevalence of scoliosis in girls
Wang et al., 1998	0	1	2				22.25	35.84	100 cases with Cobb angle <29°, 3 cases with Cobb angle between 30 and 49°, and 1 case with Cobb angle >50°
Chen, 1997							25.03	9	Low BMI may be a risk factor for scoliosis
Wang et al., 1996	12	5	12	154	25	52	39.9	368.59	There was no significance in the areas (plain, mountainous, and semi-mountainous)
Zhao et al., 1996							33.23	2.19	480 cases had Cobb angle between 5 and 39° and 7 cases had Cobb angle >40°
Yu et al., 1995							39.13	17.11	The effective rate of early physical therapy and electric stimulation for AIS was 82%

Ma et al., 1995							37.86	1158.63	81.6% cases with Cobb angle between 10 and 19°
Jiang et al., 1994							39.13	17.11	411 cases with Cobb angle between 5 and 39°, and 11 cases with Cobb angle >40°
Chen et al., 1990							35.3	57.95	25% with progression in scoliosis after one year follow, while 30% with decrease
Cao et al., 1989							42.9	647.78	The prevalence of scoliosis in Korean pupils was higher than in Han pupils. The prevalence in rural and urban areas was comparable among Korean pupils.
Zhang et al., 1988							39.9	368.59	153 cases with Cobb angle between 10 and 19°, 60 cases with Cobb angle >20°
Tan et al., 1987							22.82	196.66	There was no significance between rural and urban areas
Wang et al., 1985							32.04	25.01	Constructive scoliosis was 0.74%, while non-constructive scoliosis was 12.66%
Pin et al., 1985	3	1	0	196	10	2	28.19	170.4	A follow-up study of children who had been reported to have scoliosis at the age of 11 months showed that only half of them had scoliosis

Footnote: CS: congenital scoliosis; NMS: neuromuscular scoliosis
